# FL118, acting as a ‘molecular glue degrader’, binds to dephosphorylates and degrades the oncoprotein DDX5 (p68) to control c‐Myc, survivin and mutant Kras against colorectal and pancreatic cancer with high efficacy

**DOI:** 10.1002/ctm2.881

**Published:** 2022-05-23

**Authors:** Xiang Ling, Wenjie Wu, Ieman A. M. Aljahdali, Jianqun Liao, Sreevidya Santha, Christos Fountzilas, Patrick M. Boland, Fengzhi Li

**Affiliations:** ^1^ Department of Pharmacology & Therapeutics Roswell Park Comprehensive Cancer Center Buffalo New York USA; ^2^ Canget BioTekpharma LLC Buffalo New York USA; ^3^ Department of Cellular & Molecular Biology Roswell Park Comprehensive Cancer Center Buffalo New York USA; ^4^ Department of Medicine Roswell Park Comprehensive Cancer Center Buffalo New York USA; ^5^ Developmental Therapeutics (DT) Program Roswell Park Comprehensive Cancer Center Buffalo New York USA; ^6^ Present address: Development of Medical Oncology, Rutgers Cancer Institute of New Jersey, The State University of New Jersey, New Brunswick, NJ 08903, USA

**Keywords:** c‐Myc, colorectal cancer (CRC), DDX5/p68, FL118, human tumour animal models, mutant Kras (mKras), pancreatic ductal adenocarcinoma (PDAC), patient‐derived xenograft (PDX) tumours, survivin

## Abstract

**Background:**

Pancreatic ductal adenocarcinoma (PDAC), a difficult‐to‐treat cancer, is expected to become the second‐largest cause of cancer‐related deaths by 2030, while colorectal cancer (CRC) is the third most common cancer and the third leading cause of cancer deaths. Currently, there is no effective treatment for PDAC patients. The development of novel agents to effectively treat these cancers remains an unmet clinical need. FL118, a novel anticancer small molecule, exhibits high efficacy against cancers; however, the direct biochemical target of FL118 is unknown.

**Methods:**

FL118 affinity purification, mass spectrometry, Nanosep centrifugal device and isothermal titration calorimetry were used for identifying and confirming FL118 binding to DDX5/p68 and its binding affinity. Immunoprecipitation (IP), western blots, real‐time reverse transcription PCR, gene silencing, overexpression (OE) and knockout (KO) were used for analysing gene/protein function and expression. Chromatin IP was used for analysing protein‐DNA interactions. The 3‐[4,5‐dimethylthiazol‐2‐yl]‐2,5‐diphenyltetrazolium bromid assay and human PDAC/CRC cell/tumour models were used for determining PDAC/CRC cell/tumour in vitro and in vivo growth.

**Results:**

We discovered that FL118 strongly binds to dephosphorylates and degrades the DDX5 oncoprotein via the proteasome degradation pathway without decreasing DDX5 mRNA. Silencing and OE of DDX5 indicated that DDX5 is a master regulator for controlling the expression of multiple oncogenic proteins, including survivin, Mcl‐1, XIAP, cIAP2, c‐Myc and mutant Kras. Genetic manipulation of DDX5 in PDAC cells affects tumour growth. PDAC cells with DDX5 KO are resistant to FL118 treatment. Our human tumour animal model studies further indicated that FL118 exhibits high efficacy to eliminate human PDAC and CRC tumours that have a high expression of DDX5, while FL118 exhibits less effectiveness in PDAC and CRC tumours with low DDX5 expression.

**Conclusion:**

DDX5 is a bona fide FL118 direct target and can act as a biomarker for predicting PDAC and CRC tumour sensitivity to FL118. This would greatly impact FL118 precision medicine for patients with advanced PDAC or advanced CRC in the clinic. FL118 may act as a ‘molecular glue degrader’ to directly glue DDX5 and ubiquitination regulators together to degrade DDX5.

## Graphical abstract

- By acting as a ‘molecular glue degrader’, FL118 directly binds to and functionally dephosphorylates and degrades the multifunctional master regulator DDX5 through the proteasome degradation pathway without decreasing DDX5 mRNA.
- FL118 indirectly controls DDX5 downstream targets to inhibit cancer initiation, development, metastasis, recurrence and treatment resistance with high efficacy as demonstrated in this study using human colorectal cancer/pancreatic ductal adenocarcinoma cell and tumour models.

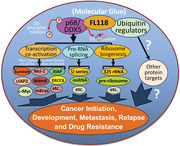

## 1 INTRODUCTION

Pancreatic ductal adenocarcinoma (PDAC) is an extremely difficult‐to‐treat cancer and is expected to become the second largest cause of cancer‐related deaths by 2030,^1^ while colorectal cancer (CRC) is the third most common cancer and the third leading cause of cancer deaths.^2^ Therefore, the development of novel and targeted molecular agents to effectively treat these cancers remains an unmet clinical need.

Precision medicine is a hallmark of modern cancer treatment, and the identification of biomarker and target is critical for clinical application to realise its precision medicine. Based on previous studies,^3^, ^4^, ^5^, ^6^, ^7^ cancer cell‐based models that stably express the survivin promoter‐luciferase reporter as a biomarker were created^8^ and used in high‐throughput screening campaigns against various compound libraries, followed by in vitro and in vivo hit‐to‐lead analyses.^9^ Thereby, a novel small molecule (named FL118) that exhibits high efficacy against advanced and treatment‐resistant CRC and PDAC tumours with favourable toxicology profiles was identified.^9^, ^10^, ^11^, ^12^, ^13^ The chemical structure of FL118 is similar to camptothecin (CPT) and its analogues, irinotecan, SN‐38 (active metabolite of irinotecan) and topotecan (Figure S1). However, FL118 uses a novel mechanism of action (MOA). CPT and its analogues use topoisomerase I (Top1) as their therapeutic target.^14^, ^15^, ^16^, ^17^ In contrast, the sensitivity of human cancer cells or tumours to FL118 is independent of Top1 expression. FL118 inhibition of cancer cell growth occurs at high pM to low nM levels, whereas FL118 inhibition of Top1 activity occurs at μM levels.^9^ FL118 can show high antitumour efficacy in human tumours with low/no Top1 expression, while tumours with high Top1 expression may exhibit insensitivity to FL118 treatment.^18^ FL118 selectively inhibits the expression of not only survivin but also Mcl‐1, XIAP and cIAP2.^9^ In contrast, SN‐38 and topotecan are 10–100‐fold weaker in the inhibition of these proteins.^9^, ^12^ Gene silencing or overexpression (OE) of these individual proteins (i.e., survivin, Mcl‐1, XIAP, cIAP2) revealed their role in FL118's effectiveness.^9^, ^11^ Furthermore, irinotecan, SN‐38 and topotecan are the substrates of the efflux pump proteins ABCG2/BCRP^19^, ^20^, ^21^, ^22^, ^23^ and Pgp/MDR1.^24^, ^25^, ^26^, ^27^, ^28^ In contrast, FL118 is not a substrate of these proteins and can bypass their expression‐induced resistance to treatment.^12^, ^29^ Consistently, FL118 effectively overcomes irinotecan and topotecan‐resistant human tumours in animal models.^12^ FL118 accumulates and resides in tumours and rapidly clears from the bloodstream (favourable pharmacokinetics).^12^ However, the mechanism by which FL118 regulates multiple cancer‐associated proteins (survivin, Mcl‐1, XIAP and cIAP2) is unknown and requires further study.

DDX5 (also called p68) is overexpressed in many types of cancer and holds great promise in molecular diagnostics, prognostics and targeted therapy.^30^ DDX5 is known to play important roles in promoting cancer‐associated gene transcription,^31^, ^32^, ^33^, ^34^, ^35^, ^36^, ^37^ miRNA expression and regulation,^38^, ^39^ pre‐mRNA splicing^40^, ^41^ and ribosome biogenesis.^42^, ^43^ DDX5 therefore plays pivotal roles in cancer development,^44^, ^45^ progression,^36^, ^46^ metastasis^47^, ^48^, ^49^ and treatment resistance.^33^, ^37^, ^44^, ^45^, ^47^, ^50^, ^51^, ^52^ DDX5 promotes cancer malignancy through multiple mechanisms, which include but may not be limited to (1) DDX5 interaction with β‐catenin to co‐activate the expression of cyclin D1 and c‐Myc^32^, ^33^, ^53^; (2) regulation of NF‐κB^35^; (3) involvement in the IncRNA, NEAT1‐mediated activation of Wnt/β‐catenin‐signalling^46^; (4) transcriptional activation of AKT^31^; and (5) coactivation of Stat3.^37^ Thus, DDX5 is considered an upstream master regulator in cancer, with great potential to become a critical target and biomarker for cancer precision medicine.^54^

In this study, we report that FL118 binds to and inhibits both the phosphorylation and expression of DDX5 (p68) in CRC and PDAC cancer cells. DDX5 is a master regulator and positively controls the expression of survivin, Mcl‐1, XIAP, cIAP2, c‐Myc and mutant Kras (mKras); thus, FL118 can further control the expression of these proteins by directly binding to and inhibiting DDX5. Silencing or knockout (KO) of DDX5 in PDAC cells resulted in significant tumour growth retardation and increased PDAC cell resistance to FL118 treatment (i.e., significant loss of FL118's antitumour activity). These findings would facilitate further elucidation of the FL118 MOA in detail and lay a foundation for further FL118 development into an innovative drug option for treating PDAC and CRC patients in the clinic.

## 2 RESULTS

### 2.1 Direct binding of DDX5 (also called p68) by FL118 with high binding affinity

**FIGURE 1:**
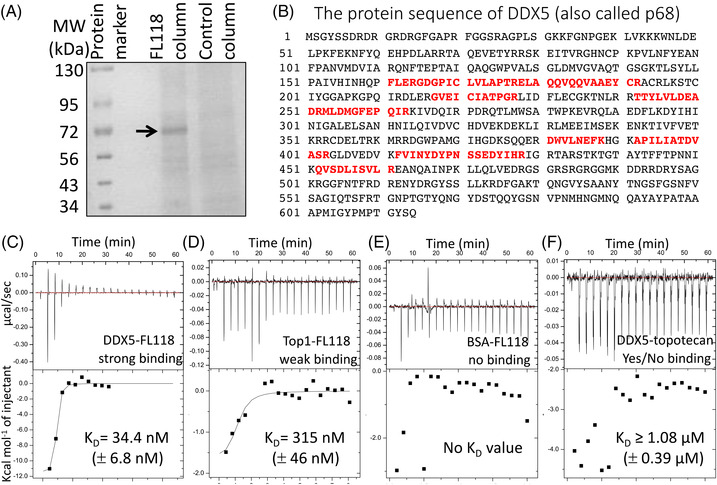
Identification of FL118 biochemical targets. Detailed methods are described in the Materials and methods section. (A) FL118 affinity column purification identified a ∼70 kD protein. FL118 was directly coupled to a beaded resin. Then, SW620 protein lysates were purified with the FL118 affinity column through steps of stringent washing, elution with 8 M urea, de‐urea, sample concentration to 20–30 μl, which was displayed on 5%–20% SDS polyacrylamide gel electrophoresis gel. (B) The ∼70 kD protein in (A) was analysed via mass spectrometry. The protein band was digested in gel, and 10 peptides were isolated and used for searching the protein database. The 10 peptides fully matched the DDX5 (also called p68) protein sequence. (C), (D), (E), (F) Representative isothermal titration calorimetry (ITC) analysis results are shown. The K_D_ values presented within the figure are the mean ± standard deviation (SD) from two ITC analyses. The y‐axis, with different labelling scales, is for result pattern visibility and dot inclusion. Purified Flag‐tagged DDX5 (Flag‐DDX5) (C, F), Flag‐Top1 (D) and bovine serum albumin (E) were loaded into a 96 DeepWell PP plate, and then FL118 (C, D, E) or topotecan (F) was automatically titrated stepwise into the protein cell by 20 injections in 60 min (one injection per 3 min) on a MicroCal‐Malvern Auto‐ITC200

Previous studies indicated that FL118 inhibits the expression of multiple antiapoptotic proteins (i.e., survivin, Mcl‐1, XIAP and cIAP2).^9^ However, the direct biochemical target of FL118 remains unknown. To find the direct protein target that can be bound by FL118, we coupled the FL118 small molecules on agarose resin beads through an immobilised linker of diaminodipropylamine (DADPA) via the ‘Mannich reaction’ (Figure S2A) to make an FL118 affinity purification column. Using the FL118 affinity column to purify whole SW620 cell‐extracted proteins, we identified an ∼70 kD protein in the FL118 column but not in the control column under the same stringent washing conditions (Figure 1A). Mass spectrometry (MS) analyses revealed that the ∼70 kD protein is DDX5/p68 (Figure 1B), a multifunctional oncogenic protein and a DEAD (Asp‐Glu‐Ala‐Asp) box RNA helicase that plays pivotal roles in a broad cancer malignancy network as summarised in the Introduction section. Furthermore, using a tritium (^3^H)‐labelled FL118 (^3^H‐FL118) as a probe, our protein microarray binding studies indicated that FL118 does not bind to other DEAD box family proteins tested, including DDX1, DDX4, DDX6, DDX10, DDX11, DDX17, DDX18, DDX19B, DDX20, DDX21, DDX25, DDX39, DDX42, DDX43, DDX47, DDX49, DDX54, DDX55 and DDX58 proteins (Table S1). Additionally, the binding of FL118 to DDX5 was alternatively determined using NANOSEP 3K OMEGA Devices with purified Flag‐tagged DDX5 (Flag‐DDX5) proteins for binding by the ^3^H‐FL118. This study indicated that FL118 binding to the DDX5 protein is about 10 times more effective than the FL118 binding to the Top1 protein (Figure S2B). Collectively, these observations indicate a reasonable FL118‐DDX5 binding specificity.

To further determine the binding affinity of FL118 with DDX5 versus Top1, Flag‐tagged DDX5 and Flag‐tagged Top1 proteins were purified using a FLAG M Purification Kit (Sigma) from their expression vector‐transfected HEK293T cells. Then, the isothermal titration calorimetry (ITC) technology was used to test the physical interactions of FL118 with DDX5 versus Top1. The binding affinity of a small molecule with a protein target usually uses the equilibrium dissociation constant (K_D_) to evaluate their interaction strengths. The smaller the K_D_ value is, the greater the binding affinity. In this regard, our ITC results revealed that FL118 strongly binds to DDX5 (K_D _= 34.4 nM, Figure 1C), which is almost 10 times stronger than the FL118 binding affinity with Top1 (K_D _= 315 nM, Figure 1D). FL118 showed no binding to bovine serum albumin (BSA, Figure 1E), while topotecan showed yes/no binding to DDX5 (K_D_ ≥ 1080 nM, Figure 1F). This result is consistent with our previous finding that FL118 antitumour activity is not dependent on Top1,^9^, ^18^ despite FL118 having a chemical structure similar to CPT, irinotecan, SN38 and topotecan (Figure S1).

### 2.2 Dephosphorylation and degradation of DDX5 by FL118 without inhibiting DDX5 mRNA

**FIGURE 2:**
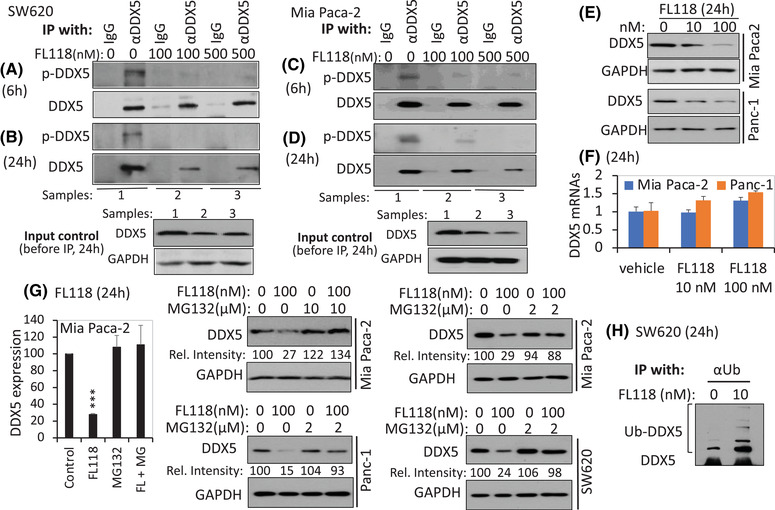
FL118 induces dephosphorylation and inhibition of the DDX5 protein. (A), (C) FL118 treatment for 6 h eliminates DDX5 phosphorylation on tyrosine (Y) residues in both SW620 cells (A) and Mia Paca‐2 cells (C). (B), (D) FL118 treatment for 24 h not only maintains the elimination of DDX5 Y phosphorylation but also inhibits DDX5 proteins in both SW620 cells (B) and Mia Paca‐2 cells (D). Colorectal cancer (CRC) SW620 cells and Pancreatic ductal adenocarcinoma (PDAC) Mia Paca‐2 cells with and without FL118 treatment for 6 h (A, C) or 24 h (B, D) as shown; cells were then analysed by immunoprecipitation (IP) with anti‐DDX5 antibody (αDDX5) or control IgG, followed by western blots with anti‐phospho‐tyrosine‐specific antibodies and αDDX5. The input controls shown in the bottom panel of (B) and (D) are 10% of cell lysates before IP. (E), (F) FL118 inhibits the DDX5 protein but not its mRNA. PDAC Panc‐1 and MiaPaca2 cells were treated with and without FL118 as shown. Cells were then analysed using western blots with DDX5 antibodies (E) or quantitative real‐time RT‐PCR (F). Data in (F) are the mean ± SD from three tests. (G) The proteasome inhibitor MG132 reverses FL118‐mediated degradation of the DDX5 protein. Mia Paca‐2, Panc‐1 and SW620 cells were treated with and without FL118 in the presence and absence of MG132 for 24 h as shown. Cells were then analysed using western blots with DDX5 antibody. The relative intensity (Rel. inten) was labelled in each western blot result. The histogram on the far‐left panel in (G) is the quantification of DDX5 protein bands (normalised to the GAPDH internal control) from two Mia Paca‐2 cell replicates. ***p value < .001. (H) FL118 treatment increases DDX5 ubiquitination. SW620 cells were treated with and without FL118 as shown. Cells were then analysed by IP with anti‐ubiquitin antibody, followed by western blots with αDDX5 antibodies. GAPDH in (B), (D), (E) and (G) is the internal protein loading control.

A critical question is whether the binding of FL118 to DDX5 shown in Figure 1 has any effect on the function and/or expression of DDX5. It has been documented that tyrosine (Y) 593/Y595‐double phosphorylated DDX5 protects glioblastoma cells from apoptosis,^52^ and Y593‐phosphorylated DDX5 promotes epithelial‐to‐mesenchymal transition (EMT) in CRC cells.^55^ To determine whether FL118 treatment could affect DDX5 phosphorylation and expression, we first performed immunoprecipitation (IP) and western blot analyses of SW620, Panc‐1 and Mia Paca‐2 cells for DDX5 phosphorylation and expression and found that Panc‐1 cells express relatively low DDX5 with minimal DDX5 phosphorylation (Figure S3). Therefore, we used SW620 and Mia Paca‐2 cells in the studies. We found that FL118 binding to DDX5 rapidly induced the tyrosine dephosphorylation of DDX5 without a DDX5 protein decrease at 6 h in both SW620 cells (Figure 2A) and Mia Paca‐2 cells (Figure 2C). However, after SW620 cells and Mia Paca‐2 cells were treated with FL118 for 24 h, DDX5 protein decreased, while DDX5 dephosphorylation status could be maintained (Figure 2B,D). Intriguingly, the FL118‐mediated DDX5 protein decrease (Figure 2E) was not accompanied with decreasing DDX5 mRNA (Figure 2F). Furthermore, consistent with these findings, the FL118‐induced degradation of DDX5 protein in Mia Paca‐2, Panc‐1 and SW620 cells can be reversed in the presence of the proteasome inhibitor MG132 (Figure 2G). Additionally, FL118 treatment increased the ubiquitination (Ub) of DDX5 even in the absence of MG132 (Figure 2H). These observations suggest that FL118‐induced DDX5 degradation occurs through the ubiquitin‐proteasome degradation pathway.

### 2.3 Control of survivin, Mcl‐1, XIAP, cIAP2, c‐Myc and mKras by DDX5

**FIGURE 3:**
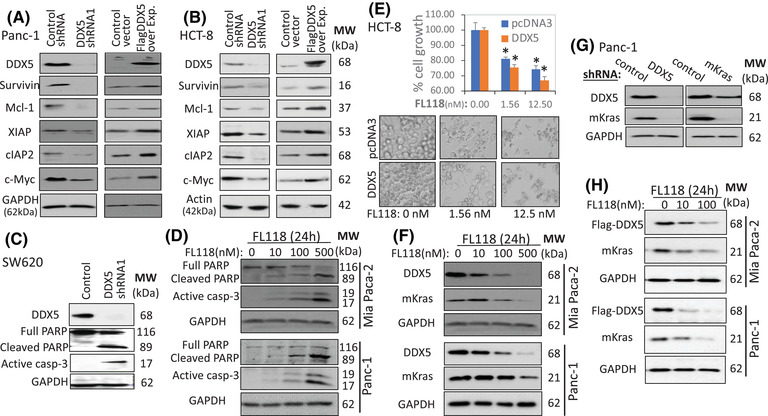
Genetic modulation of DDX5 in either PDAC or CRC cells results in the modulation of FL118‐inhibiting proteins. (A), (B) (left panels), shRNA silencing of DDX5 decreases the expression of survivin, Mcl‐1, XIAP, cIAP2 and c‐Myc. Cells were infected with control shRNA or DDX5 shRNA as shown. Infected cells were lysed 48 h post infection; the cell lysates were used to determine the expression of DDX5, survivin, Mcl‐1, XIAP, cIAP2 and c‐Myc through western blots. (A), (B) (right panels), Forced expression of Flag‐DDX5 increases the expression of survivin, Mcl‐1 XIAP, cIAP2 and c‐Myc. Cells were transfected with empty vector (control) or Flag‐DDX5 expression vectors (RC200371, OriGene) using Lipofectamine 2000. Cells were lysed 48 h post‐transfection and subjected to western blot analysis using their corresponding antibodies. Overexpressed Flag‐DDX5 was detected using the Flag antibody in (A); endogenous DDX5 could not be detected in this case (A, right panel). Overexpressed Flag‐DDX5 was detected using DDX5 antibody in (B); in this case, Flag‐DDX5 protein is slightly larger (B, right panel). (C) Silencing of DDX5 induces apoptosis: SW620 cells were infected with control lentiviral particles or lentiviral DDX5 shRNA particles. Infected cells were lysed 48 h post infection to test caspase‐3 activation and PARP cleavage (a hallmark of apoptosis) using western blots. (D) FL118 treatment activates apoptosis hallmarks in Mia Paca‐2 and Panc‐1 cells. (E) Forced Flag‐DDX5 expression sensitises cells to FL118. Flag‐DDX5‐overexpressing HCT‐8 cells were treated with FL118 for 72 h. Cells were then analysed using the MTT assay after images were taken under each condition. Each bar is the mean ± SD derived from three tests (top panel). Each image is a representative example for each condition with and without FL118 treatment, as shown. (F) FL118‐inhibiting DDX5 is associated with mutant Kras (mKras) inhibition. MiaPaca2 and Panc‐1 cells with and without FL118 treatment for 24 h are shown. Cells were then analysed using western blots with corresponding antibodies. (G) Knockdown of DDX5 abrogates mKras. Panc1 cells were infected with lentiviral particles containing control shRNA, DDX5 shRNA or mKras shRNA. Infected cells were lysed and analysed for the expression of DDX5 and mKras using western blots 48 h post infection. (H) FL118 could inhibit Flag‐DDX5 expression driven by a heterologous CMV promoter, which was also associated with mKras decrease. Mia Paca‐2 and Panc‐1 cells were transfected with CMV‐driven Flag‐DDX5. After cells were selected with kanamycin, the selected transfectants were treated with and without FL118 for 24 h as shown. Cells were then determined for the expression of Flag‐DDX5 and mKras using antibodies for Flag and Kras. GAPDH in (A), (C), (D), (F), (G), (H) and actin in (B) are internal total protein loading controls.

Our previous studies indicated that FL118 inhibits the expression of survivin, Mcl‐1, XIAP and cIAP2.^9^ To gain insight into the relationship of the FL118‐binding DDX5 with the FL118 inhibition of survivin, Mcl‐1, XIAP and cIAP2, we silenced and overexpressed DDX5 in PDAC Panc1 (Figures 3A and S4A) and CRC HCT‐8 (Figures 3B and S4B) cells. These studies revealed that silencing of DDX5 using lentiviral DDX5‐specific shRNA (Figure S5) downregulates survivin, Mcl‐1, XIAP, cIAP2 and c‐Myc (Figures 3A and S4A, left panel), while forced expression of DDX5 upregulates these proteins (Figures 3A and S4A, right panel). Similar results were obtained in CRC cells (Figures 3B and S4B). Given that SW620 cells highly express DDX5 (Figure S3), shRNA silencing of DDX5 in SW620 cells induces apoptosis hallmarks (PARP cleavage and caspase‐3 activation, Figure 3C), which mimics the effect of the FL118 treatment in both Mia Paca‐2 and Panc‐1 cells (Figure 3D). In contrast, forced expression of DDX5 in HCT‐8 cells (expressing moderate DDX5) enhances FL118‐mediated cell growth/viability inhibition (Figure 3E, upper panel histogram) and cell death (Figure 3E, lower panel image). These observations are fully consistent with our previous finding indicating that FL118 inhibits the expression of survivin, Mcl‐1, XIAP and cIAP2 and induces apoptosis in cancer cells.^9^, ^13^, ^56^

It is well known that mutation‐activated, that is, mKras is a critical target for PDAC treatment, although it is difficult to target. Intriguingly, our studies revealed that the inhibition of DDX5 by FL118 is associated with mKras inhibition in both Mia Paca‐2 and Panc‐1 cells (Figure 3F). To study the potential role of DDX5 in the control of mKras expression, we used genetic approaches to independently knock down each of the genes using their shRNA to determine the other gene expression. Our studies indicated that shRNA silencing of DDX5 completely abrogated mKras expression, while shRNA silencing of mKras only weakly reduced DDX5 expression (Figure 3G). These results clearly indicate that mKras is also a downstream target of DDX5. Furthermore, forced expression of Flag‐DDX5 in Mia Paca‐2 and Panc‐1 cells could also be inhibited by FL118 as well and associated with mKras decrease (Figure 3H). These observations not only further indicate that mKras is a downstream target of DDX5 but are also consistent with the finding that the inhibition of DDX5 by FL118 occurs through the direct induction of DDX5 degradation by FL118 (Figure 2G,H).

Together, these studies indicate that DDX5 acts as an upstream master regulator to control the expression of survivin, Mcl‐1, XIAP, cIAP2, c‐Myc and mKras, which are key oncogenic proteins involved in cancer development and malignant networks.

### 2.4 In**hibition of survivin transcription by FL118 through the FL118‐DDX5‐c‐Myc‐CDK9‐cyclin T1‐survivin pathway**

**FIGURE 4:**
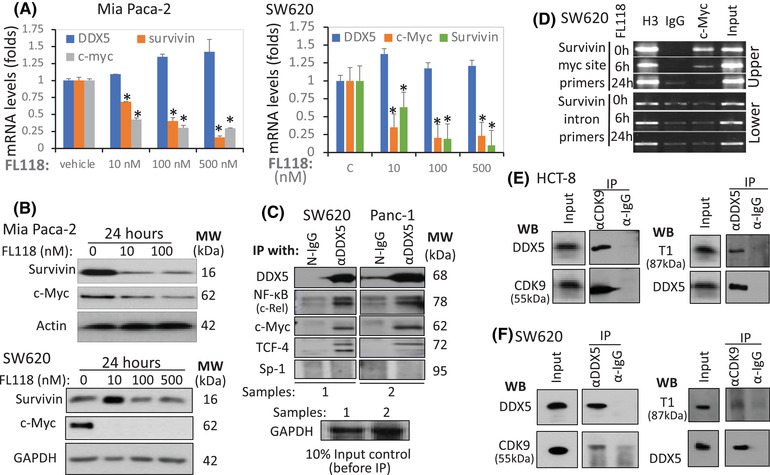
Relationship of FL118 with DDX5, c‐Myc and survivin. (A) FL118 inhibits both survivin and c‐Myc mRNA expression. (B) FL118 inhibits both survivin and c‐Myc protein expression. MiaPaca2 and SW620 cells were treated with vehicle or FL118 for 24 h, the mRNA (A) or protein (B) expression of DDX5, survivin and c‐Myc was determined by real‐time RT‐PCR (A) or western blots (B). Each bar in A is the mean ± SD from three tests. Actin and GAPDH in (B) are internal protein loading controls. (C) DDX5 interacts with c‐Rel, c‐Myc and TCF‐4 but not Sp‐1. SW620 and Panc‐1cells were analysed by IP with (αDDX5, followed by western blots with antibodies for DDX5 (control), c‐Rel, c‐Myc, TCF4 and Sp‐1. IP with corresponding normal IgG (N‐IgG) was used as the internal control. The bottom panel in (C) is the input controls (10% of cell lysates before IP). (D) FL118 can abrogate c‐Myc from the survivin promoter c‐Myc binding site: A SimpleChIP Enzymatic Chromatin IP (ChIP) Kit was used in the ChIP assay with primers covering the c‐Myc binding site in the survivin promoter (upper panel) or with primers from the survivin intron region (lower panel, negative control). Histone 3 (H3) binding is a positive control. (E) DDX5 interacts with both CDK9 and T1 in CRC HCT‐8 cells. Cells were lysed and immunoprecipitated with CDK9 antibody or control IgG, followed by western blots with DDX5 antibody (left upper panel) or CDK9 antibody (left lower panel, control). The same cell lysates were immunoprecipitated with DDX5 antibody or control IgG, followed by western blots with T1 antibody (right upper panel) or DDX5 antibody (right lower panel, control). The input control was 10% of cell lysates before IP. (F) DDX5 interacts with both CDK9 and T1 in CRC SW620 cells. Cells were lysed and immunoprecipitated with CDK9 antibody or control IgG, followed by Western blots with DDX5 antibody (left upper panel) or CDK9 antibody (left lower panel, control). The same cell lysates were immunoprecipitated with DDX5 antibody or control IgG, followed by Western blots with T1 antibody (right upper panel) or DDX5 antibody (right lower panel, control). The input control is 10% of cell lysates before IP.

It has been documented in previous studies that DDX5 acts as a transcription co‐activator to promote oncogene transcription through interactions with other transcription factors (TFs). For example, DDX5 interacts with β‐catenin and regulates cyclin D1 and c‐Myc^33^, ^53^; DDX5 regulates NF‐κB^35^ and co‐activates Stat3.^37^ Consistent with these documented studies and the data shown in Figures 1, 2, 3, FL118 inhibits both mRNA and protein of c‐Myc and survivin without inhibiting DDX5 mRNA in both Mia Paca‐2 and SW620 cells (Figure 4A,B). This is consistent with our previous studies, which showed that FL118 selectively inhibits survivin promoter activity.^9^

Next, we studied the mechanism of DDX5‐mediated transcriptional control of the survivin gene. We first investigated the interactions of DDX5 with the relevant TFs, c‐Rel (NF‐κB), c‐Myc and TCF‐4 together with the universal TF Sp‐1. Our studies indicated that DDX5 interacts with c‐Rel/NF‐κB, c‐Myc and TCF4 but not Sp‐1 (Figure 4C). Since the survivin core promoter has a c‐Myc binding site^57^ and also FL118 downregulates c‐Myc expression (Figure 4B), we then determined whether FL118 could affect c‐Myc binding to the survivin promoter at the c‐Myc binding site. Using the survivin promoter's c‐Myc DNA‐binding site primers‐mediated Chromatin IP (ChIP) assay, we found that in comparison with the FL118‐untreated control (Figure 4D, upper panel, top lane), FL118 treatment for 6 h significantly decreased c‐Myc binding on the survivin promoter (Figure 4D, upper panel, middle lane), while FL118 treatment for 24 h completely removed the c‐Myc binding on the survivin promoter (Figure 4D, upper panel, bottom lane), which is consistent with the data in Figure 4B. In contrast, the survivin gene intron DNA primer‐mediated ChIP assay as the negative control showed no such changes under the same experimental conditions (Figure 4D, lower panel).

It was reported that CDK9 and cyclin T1 (T1), as the core component of positive transcriptional elongation (TE) factor b (P‐TEFb), interact with c‐Myc,^58^, ^59^, ^60^, ^61^ and DDX5 interacts with CDK9.^62^, ^63^ Consistent with these documented findings, our studies using IP and western blots indicated that DDX5 interacts with both CDK9 and T1 (Figure 4E,F). It has been documented that CDK9/T1 controls extensive crosstalk between TFs and the RNA polymerase II (RNAPII) complex for efficient transcription of required mRNA.^64^, ^65^, ^66^ Our studies indicated that one strategy for FL118 to block the crosstalk of DDX5‐c‐Myc with the RNAPII complex on the survivin core promoter is that FL118 alone could inhibit both CDK9 and cyclin T1 proteins (Figure S6A), which can be partially rescued by adding hexamethylene bisacetamide (HMBA, a P‐TEFb activator) or HMBA plus MG132. This suggests that the inhibition of CDK9 and T1 by FL118 is also post‐translationally regulated and may involve the protection of FL118‐mediated degradation of CDK9 and T1 by HMBA and MG132 blocking the proteasome degradation pathway, which needs further investigation. Nevertheless, our studies revealed a novel MOA for FL118 to inhibit survivin transcription through the FL118‐mediated inhibition of the DDX5‐c‐Myc‐CDK9/T1 pathway. This is significant because the participation of P‐TEFb in transcriptional controls is critical for the transition of the RNAPII complex from its stalling state into its elongation state.^65^, ^67^, ^68^ Thus, the DDX5‐c‐Myc‐CDK9/T1 signalling could interact with the basic transcription machinery on the survivin promoter to control the survivin gene transcription (Figure S6B). Additionally, it is possible that FL118‐mediated inhibition of multiple DDX5 downstream targets may employ a similar MOA as determined here, for the example of the survivin gene since, as shown in a previous publication,^57^ these DDX5 target genes have highly overlapped TFs. Furthermore, although P‐TEFb is a general co‐activator, its MOA on a particular gene can be gene‐specific.^69^

### 2.5 Association of high DDX5 with high sensitivity of PDAC and CRC tumours to FL118

**FIGURE 5:**
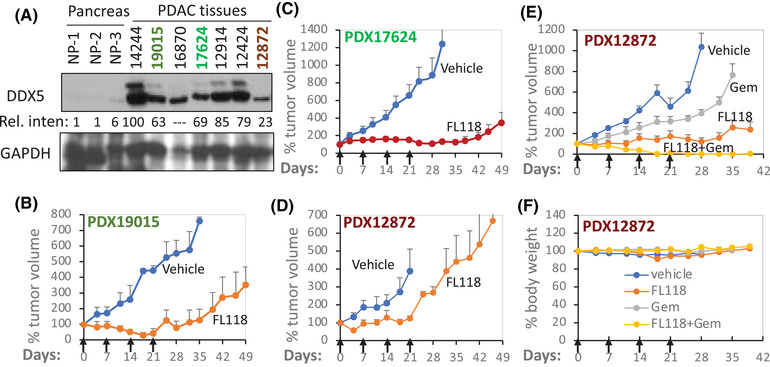
DDX5 expression is enhanced in PDAC, and PDAC tumours with high DDX5 expression are linked to high sensitivity to FL118. (A) Expression of DDX5 in three human normal pancreases and seven PDAC tumour specimens is analysed using western blots. GAPDH is used as the internal control. The Rel. inten of the western blot bend for DDX5 in each tumour tissue was added by setting the 14244 tumour DDX5 expression as 100 after being normalised to the GAPDH internal control. Of note, since the GAPDH expression is abnormal in the 16870‐tumour tissue, quantification of this tumour tissue was not assessed. (B), (C), (D) patient‐derived xenograft (PDX; PDX19015 (B), PDX17624 (C) and PDX12872 (D) tumour growth curves after vehicle and FL118 treatment. The PDX tumour model setup and treatment are described in the Methods section. Treatment with vehicle or FL118 was weekly x 4 by oral administration (arrowed) at 2.5 mg/kg (1/4 MTD). (E) PDX12872 tumour growth curves after vehicle and FL118 treatment alone or in combination with gemcitabine (Gem, FL118, 5 mg/kg (1/2 MTD), orally; Gem, 40 mg/kg, intraperitoneally, weekly x 4, arrowed). (F) Mouse body weight changes from the (E) study. Each tumour curve (B, C, D, E) or each mouse body weight change curve (F) is the mean + SD from five mice.

Data from the Human Protein Atlas Database have documented that DDX5 expression is enhanced in 83% of PDAC tumours (Figure S7). Consistently, western blot analyses of three available normal pancreases (NPs) and seven PDAC tissues for establishing patient‐derived xenograft (PDX) tumours indicated that while none of the three NP expressed DDX5, all seven of the PDAC tumours expressed DDX5 from high (PDX14244) to low (PDX12872) levels (Figure 5A). Consistent with the high expression of DDX5 in the PDAC PDX14244, our previous studies demonstrated that PDX14244 tumours have high sensitivity to FL118 and can be eliminated by FL118 treatment at half maximum tolerated dose (1/2 MTD) with only one cycle (weekly x 4).^13^ Here, we further show that PDAC PDX19015 and PDX17624 tumours with moderate expression of DDX5 were regressed after one cycle of FL118 treatment (Figure 5B,C). In contrast, the PDAC PDX12872 tumours with a low‐level DDX5 expression (Figure 5A) exhibited less sensitivity to FL118 treatment (Figure 5D). However, as with the previously reported case of the PDAC PDX10978 tumour,^13^ the PDX12872 tumours can be effectively eliminated by FL118 in combination with a low level of gemcitabine (Figure 5E) at non‐toxic dose levels (Figure 5F). This demonstrated an intriguing approach for FL118 to treat PDAC/CRC tumours with low DDX5 expression. This finding is also significant because some patients in their tumours with low DDX5 expression can be treated with FL118 as well, with a low level of chemotherapeutics.

**FIGURE 6:**
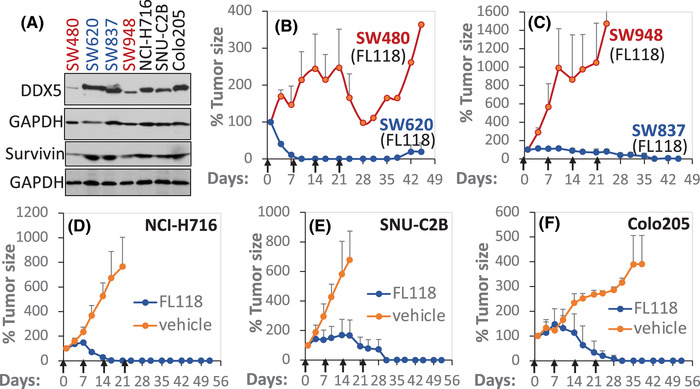
CRC tumours with high DDX5 link to high FL118 sensitivity in vivo. (A) DDX5 and survivin expression is determined using western blots in seven CRC cell lines as shown. GAPDH is the internal control. (B), (C) Comparison of DDX5 low (SW480, SW948) versus DDX5 high (SW620, SW837) CRC tumours’ sensitivity to FL118 treatment. To simplify Figure 2B,C, we removed the vehicle‐treated SW480, SW948, SW620 and SW837 tumour curves. Within 3 weeks, these vehicle‐treated tumours grew into the largest tumour size allowed, and the tumour mice were euthanised according to the Institutional Animal Care and Use Committee regulation. (D), (E), (F) FL118 sensitivity in additional three CRC cell line‐established tumours (NCI‐H716, SNU‐C2B, Colo205) in SCID mice. Each CRC cell line (2 × 10^6^ per tumour site) was subcutaneously injected into 2–3 SCID mice in the flank area to establish xenograft tumours. SCID mice with the established tumour were used for planned experimental studies. The experimental tumour model set up from human tumour‐maintained mice is described in the Section 4.18. Treatment with vehicle or FL118 at 5 mg/kg (1/2 MTD) was weekly x 4 via oral administration (arrowed). Each tumour curve is the mean tumour size ± SD from five SCID mice.

Similarly, the data from the Human Protein Atlas Database also documented that DDX5 expression enhances in all 11 CRC tumours (high: 5; medium: 6; low: 0). Our studies of three available pairs of clinical non‐tumour adjacent (Non) and CRC tumour (Tu) specimens using western blot analyses indicated that two of the three CRC tumours showed enhanced DDX5 expression (Figure S8). Significantly, consistent with DDX5 acting as a master regulator to control a panel of oncogenic proteins, as shown in Figure 3A,B, CRC cells with high DDX5 are linked to high survivin, and those with low DDX5 are linked to low survivin (Figures 6A and S8). Furthermore, as seen in the PDAC PDX tumours shown in Figure 5, CRC tumours with high DDX5 show high sensitivity to FL118 treatment (Figure 6B–F), while SW480 and SW948 tumours with low DDX5 expression show poor sensitivity to FL118 (Figure 6B,C, red curves). Together, the in vivo data from both PDAC (Figure 5) and CRC (Figure 6) tumours strongly support that the FL118's target, DDX5, can act as a biomarker and target for predicting PDAC and CRC tumour sensitivity to FL118 treatment. This is crucial to the design of future biomarker and target‐driven FL118 clinical trials for PDAC and CRC patients.

### 2.6 Retardation of tumour growth by DDX5 knockdown and enhancement of FL118 efficacy by DDX5 OE

**FIGURE 7:**
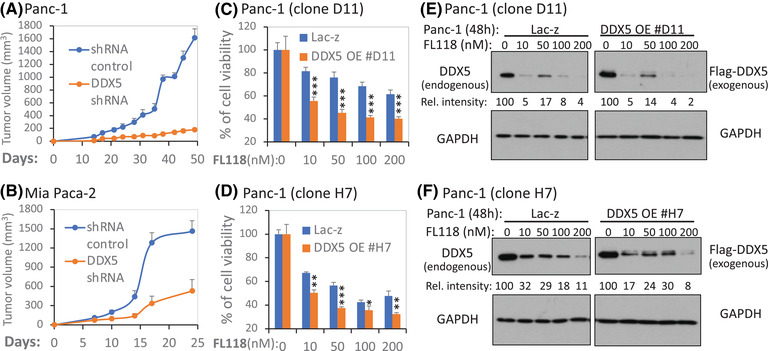
Genetic modulation of DDX5 in PDAC cells affects tumour growth, cell viability and FL118 responsiveness. (A), (B) Silencing of DDX5 delays PDAC tumour growth: Control and DDX5‐specific shRNA lentiviral particle‐infected cells (2 × 10^6^) were subcutaneously injected into each site in the flank area of SCID mice. Tumour growth was monitored over time. The tumour growth curve from each time point is the mean ± SD from 5 tumours from five mice. (C), (D) Overexpression (OE) of DDX5 increases FL118 efficacy to inhibit PDAC cell growth/viability: Two DDX5 OE Panc‐1‐cell clones (C, clone #D11; D, clone #H7) in parallel with Lac‐z control Panc‐1‐cell clones were treated with and without FL118 as shown for 72 h. Cell viability was then determined using the MTT assay. The data are the mean ± SD derived from three tests. *p < .05; **p < .01; ***p < .001. (E), (F) FL118 could degrade both endogenous and exogenous DDX5. Panc‐1 D11 and H7 cloning cells that were forced to express Lac‐z (control) or Flag‐DDX5 were treated with and without FL118 treatment as shown for 48 h. Cells were then analysed using western blots with DDX5 antibodies (E, F, left panel) or with Flag antibodies (E, F, right panel). GAPDH was used as the internal control for total protein loading. The relative (Rel.) intensity of the western blot bands in each lane for DDX5 expression was provided by setting the band without FL118 treatment as 100 after being normalised to the GAPDH internal control

So far, our data presented above have demonstrated that (1) DDX5 is a direct physical and functional target of FL118 (Figures 1 and 2) and (2) genetic manipulation of DDX5 expression can affect the expression of a panel of oncogenic and antiapoptotic proteins (Figure 3). This likely occurs through DDX5‐mediated transcription, which was demonstrated using the survivin gene as an example (Figure 4); and (3) DDX5 appears to be a biomarker for reflecting PDAC and CRC tumour sensitivity to FL118 treatment (Figures 5 and 6). Next, we investigated whether the modulation of DDX5 expression could affect tumour cell growth and sensitivity to FL118 treatment. Our studies indicated that DDX5‐silenced PDAC Panc‐1 and Mia Paca‐2 cells exhibited slower tumour formation and growth than the corresponding control shRNA PDAC cells after their implantation into the flank area of SCID mice (Figure 7A,B). In contrast, overexpresson (OE) of DDX5 (i.e., provides more FL118 targets) enhances FL118 efficacy to inhibit PDAC cell viability (Figure 7C,D). Based on our experiences from past studies and given that DDX5 is a direct target of FL118, high DDX5 showing more sensitivity to FL118 is likely due to the situation that after cells with DDX5 OE were obtained through a process of cell clone selection, these DDX5 OE cell clones that could be successfully selected are likely those that have a growth advantage and exhibit greater addiction to DDX5 for survival; thus, FL118‐mediated degradation of DDX5 would exhibit higher FL118 efficacy. Consistent with this notion, our data shown in Figure 7E,F indicated that FL118 could effectively degrade both endogenous and exogenous DDX5, which matches the functional data shown in Figure 7C,D. Together, these observations further support the findings that DDX5 is the direct biochemical target of FL118 and is a master regulator of cancer cell and tumour survival and growth.

### 2.7 Significant loss of FL118 anticancer activity upon DDX5 KO in PDAC cells

**FIGURE 8:**
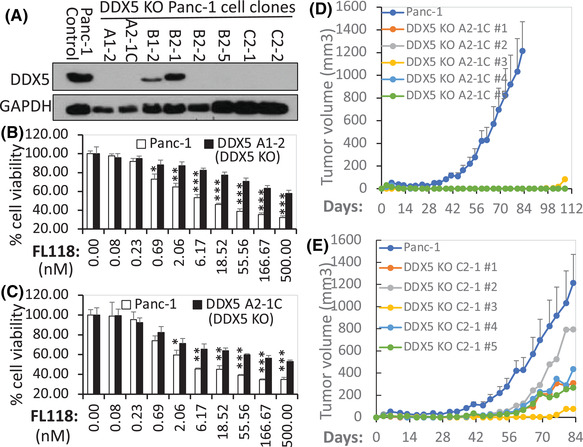
(A) Detection of DDX5 knockout (KO) cell clones. The expression profile of DDX5 in various Panc‐1 individual cell clones in parallel with the parental Panc‐1 control cells analysed by western blots is shown. GAPDH was used as the internal control. (B), (C) DDX5 KO in PDAC Panc‐1 cells results in FL118 loss of function to inhibit cell growth/viability: DDX5 KO Panc‐1 clone A1‐2 cells (B) and DDX5 KO Panc‐1 clone A2‐1C cells (C) in parallel with control Panc‐1 cells (B, C) were treated with and without FL118 as shown. Cell viability was determined by MTT assay 72 h after with and without FL118 treatment. Each bar is the mean ± SD derived from three assays. *p < .05; **p < .01; ***p < .001. (D), (E) In vivo tumour formation and growth of DDX5 KO Panc‐1 cells and corresponding control cells are shown: DDX5 KO Panc‐1 clone A2‐1C cells (2 × 10^6^, D) and DDX5 KO Panc‐1 clone C2‐1 cells (2 × 10^6^, E) in parallel with the parental Panc‐1 control cells (2 × 10^6^, D, E) were subcutaneously injected into each site at the flank area of SCID mice. Tumour growth was monitored over time. The parental Panc‐1 control cell tumour growth curve at each time point is the mean ± SD from five tumours from five mice. The DDX5 KO cell tumour growth curves are the individual tumour growth curves derived from five tumours from five mice.

**FIGURE 9:**
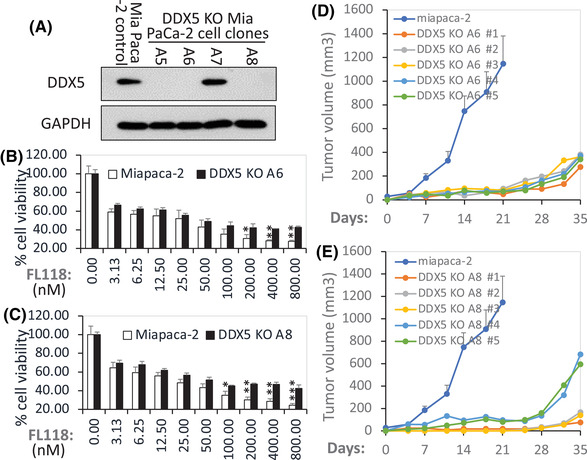
(A) Detection of DDX5 KO cell clones. The expression profile of DDX5 in various Mia Paca‐2 individual cell clones in parallel with the parental Mia Paca‐2 control cells analysed by western blots is shown. GAPDH was used as the internal control. (B), (C) DDX5 KO in Mia PaCa‐2 cells results in FL118 loss of function to inhibit cell growth/viability: DDX5 KO Mia Paca‐2 clone A6 cells (B) and DDX5 KO Mia Paca‐2 clone A8 cells (C) in parallel with parental Mia Paca‐2 control cells (B, C) were treated with and without FL118 as shown. Cell viability was determined by MTT assay 72 h after with and without FL118 treatment. Each bar is the mean ± SD derived from three assays. *p < .05; **p < .01; ***p < .001. (D), (E) In vivo tumour formation and growth of DDX5 KO Mia Paca‐2 cells and corresponding control cells are shown: DDX5 KO Mia Paca‐2 clone A6 cells (2 × 10^6^, D) and DDX5 KO Mia Paca‐2 clone A8 cells (2 × 10^6^, E) in parallel with the parental Mia Paca‐2 control cells (2 × 10^6^, D, E) were subcutaneously injected into each site in the flank area of SCID mice. Tumour growth was monitored over time. The parental Mia Paca‐2 control cell tumour growth curve at each time point is the mean ± SD from five tumours from five mice. The DDX5 KO cell tumour growth curves are the individual tumour growth curves derived from five tumours from five mice.

Next, we used Crispr‐Cas9 technology to knock out DDX5 in PDAC cells. To simplify the DDX5 knockout (KO) process, we directly used the vector‐free DDX5 sgRNA‐Cas9 enzyme ribonucleoprotein (RNP) via electroporation approaches for DDX5 gene KO in PDAC cells. This not only makes DDX5 gene KO a one‐step process but also automatically leads to the RNP vanishing from the cells after a few days in cell culture; thus, the parental cells are perfect control cells for the DDX5 KO cells. Through the PCR validation of the DDX5 KO cell pool (Figure S9), followed by single‐cell cloning and the validation of individual DDX5 KO cell clones via western blot analyses, we were able to obtain multiple cell clones without DDX5 from both the Panc‐1‐cell line (Figure 8A) and the Mia Paca‐2 cell line (Figures 9A and S10). To determine whether Panc‐1 cells without DDX5 can cause cells to be less sensitive to FL118‐inhibited cell viability, parental control Panc‐1 cells and DDX5 KO Panc‐1 cells were treated with a series of FL118 concentrations for 72 h; cell growth/viability inhibition by FL118 was then determined using the 3‐[4,5‐dimethylthiazol‐2‐yl]‐2,5‐diphenyltetrazolium bromid (MTT) assay. The results indicated that DDX5 KO Panc‐1 cells significantly increased resistance to FL118 treatment (Figure 8B,C). Furthermore, the tumour formation and growth from the parental control Panc‐1 cells versus the DDX5 KO Panc‐1 cells were further compared in animal models. Our studies indicated that DDX5 KO cells significantly delayed tumour formation and growth (Figure 8D,E).

To determine whether Mia Paca‐2 cells without DDX5 can also cause cells to be less sensitive to FL118‐inhibited cell growth/viability, parental control Mia Paca‐2 cells and DDX5 KO Mia Paca‐2 cells were treated with a series of FL118 concentrations for 72 h; cell growth/viability was then determined using the MTT assay. Similar results to the DDX5 KO Panc‐1‐cell clones (Figure 8) were obtained from the DDX5 KO PDAC Mia Paca‐2 cells (Figure 9). We noticed that different DDX5 KO PDAC cell clones exhibited different tumour formation abilities. For example, the A2‐1C clone tumour formation shown in Figure 8D is much slower than the C2‐1 clone tumour formation shown in Figure 8E. Such variations in different DDX5 KO clones suggest PDAC cell heterogeneity and the varying importance of DDX5 in different PDAC cells. Additionally, the same DDX5 KO clone can also exhibit different tumour growth rates. For example, the C2‐1 clone tumour in different individual mice exhibited different tumour growth rates (Figure 8E), even though it was the same DDX5 KO cell clone (C2‐1). These variations in the same DDX5 KO clone suggest that individual mice can have different tumour formation and growth acceptability (tumour‐host effects). We will discuss in detail such variations in the third part (Section 3.3) of the Discussion section.

Nevertheless, the loss of FL118 anticancer activity after DDX5 KO in PDAC cells (Figures 8 and 9) is consistent with the data showing that DDX5 is a physical and functional FL118 target and biomarker as shown in the data from Figures 1, 2, 3, 4, 5, 6, 7. Additionally, we used the DDX5 lentiviral particles to reconstitute the DDX5 KO Mia Paca‐2 A6 cells; then, we determined whether the DDX5‐reconstituted A6 cells can be re‐sensitised to FL118‐mediated inhibition of cell growth/viability. Our preliminary results indicated that the DDX5‐reconstituted A6 cells are more sensitive to FL118 treatment than the DDX5 KO A6 cells (Figure S11).

## 3 DISCUSSION

Drug discovery expert Robert M. Plenge has indicated that ‘a good drug is one that binds to and modulates a molecular target in such a way that is safe and effective in the disease context for which it is administered’.^70^ Based on the data presented in this report, we believe that the anticancer small molecule FL118 fits this definition well. We will now discuss our data, its implications and its application for the treatment of PDAC and CRC in the clinic in the following three subsections.

### 3.1 FL118 biochemical target and functional relevance

We have demonstrated the uniqueness of the FL118 MOA in our previous studies.^57^, ^71^, ^72^, ^73^ In this study, we reported the discovery of the FL118 physical and functional target and further strengthened our knowledge and understanding of the FL118 MOA. Through FL118‐conjugated agarose resin chromatography affinity purification and alternative confirmation, we demonstrated that FL118 strongly binds to the multifunctional oncogenic protein DDX5 with high affinity (Figure 1), while FL118 does not bind to other tested DEAD (Asp‐Glu‐Ala‐Asp) box family proteins (Table S1). Significantly, the physical binding of FL118 to DDX5 is of high functional relevance. FL118 binding to DDX5 rapidly and sustainedly abrogated the tyrosine (Y) phosphorylation of DDX5 (Figure 2A–D). This is significant because it has been shown that Y‐phosphorylated DDX5 is involved in promoting cell proliferation and cancer development,^44^ EMT (pY593‐DDX5)^55^ and treatment resistance (pY593‐DDX5 and pY595‐DDX5).^52^ Furthermore, following the rapid tyrosine dephosphorylation of DDX5 by FL118 treatment, FL118 subsequently induced DDX5 degradation without inhibitory effects on DDX5 mRNA (Figures 2B,D–F and 4A) while sustaining the dephosphorylation status of DDX5 over time (Figure 2A–D). DDX5 degradation by FL118 appears to employ the ubiquitin‐mediated proteasome degradation pathway because in the presence of the proteasome inhibitor MG132, FL118‐mediated DDX5 protein degradation could be restored (Figure 2G). Consistently, DDX5 Ub was detected after FL118 treatment even in the absence of MG132 (Figure 2H). The relatively low poly‐Ub profile shown in Figure 2H is consistent with the process by which poly‐ubiquitinated DDX5 is rapidly degraded via the protein Ub degradation pathway over time after FL118 treatment in the absence of a proteasome inhibitor. To the best of our knowledge, FL118 is the first small molecule that has dual roles as an inhibitor of DDX5 Y phosphorylation and an inducer of DDX5 protein degradation. While FL118 acts like the well‐known proteolysis‐targeting chimera (PROTAC)^74^ to degrade DDX5, FL118 is more than just a DDX5 degrader and has additional advantages over PROTAC, including its novel mechanism‐based potentially favourable physicochemical properties, which may not need a ligandable pocket on the DDX5 target by directly acting as a ‘molecular glue degrader’.^75^ As pointed out by Dong et al. in their recent review article, ‘molecular glue degraders have the advantages of degrading unligandable proteins by promoting extensive network interactions between ligase and target far beyond what is attainable by small molecules alone’.^75^ Therefore, the finding reported in this study is significant and would lay a foundation for molecular cancer precision medicine and therapeutics by using DDX5 as an FL118 target and biomarker. However, while we have successfully identified and determined that DDX5 is a physically and functionally relevant target of FL118, further studies are recommended in terms of elucidating proteins that were involved in the Y dephosphorylation, Ub and degradation of the DDX5 protein upon FL118 treatment.

### 3.2 DDX5 downstream targets and FL118‐DDX5 regulation of the survivin gene transcription

FL118 was initially discovered by using a survivin promoter‐driven luciferase reporter in genetically engineered cancer cell models via compound library high‐throughput screening.^8^, ^9^ As shown in the literature, a major function of DDX5 is to act as a transcriptional co‐activator to regulate gene transcription through multiple pathways. For example, (i) DDX5 interaction with β‐catenin to regulate cyclin D1 and c‐Myc expression^33^, ^53^; (ii) DDX5 regulation of NF‐κB^35^; (iii) DDX5 transcriptional activation of AKT^31^ and (iv) DDX5 coactivation of Stat3.^37^ Additionally, our previous studies indicated that FL118 inhibits the expression of multiple antiapoptotic proteins (survivin, Mcl‐1, XIAP, cIAP2) while inducing proapoptotic proteins (Bad, Bim or Bax).^9^, ^13^ Given these documented studies as well as the binding and functional relevance of FL118 to DDX5 (Figures 1 and 2), we thought that DDX5 may also regulate survivin, Mcl‐1, XIAP and cIAP2 as its novel downstream targets. Consistent with this logic, our studies found that the silencing of DDX5 using DDX5‐specific shRNA (Figure S5) either in PDAC cells or in CRC cells resulted in decreased expression of survivin, Mcl‐1, XIAP, cIAP2 and c‐Myc (a known DDX5 target; Figure 3A,B, left panels). Silencing DDX5 was associated with apoptotic marker induction (Figure 3C) and mimicked FL118 treatment (Figure 3D). In contrast, forced expression of DDX5 using DDX5 expression vectors resulted in increased expression of survivin, Mcl‐1, XIAP, cIAP2 and c‐Myc (Figure 3A,B, right panels). In line with the fact that DDX5 is a direct physical and functional target of FL118, forced expression of the DDX5 targets in CRC cells (which will make cells more addictive to DDX5 for survival) increased FL118 effectiveness to inhibit cancer cell growth/viability and death (Figures 3E and 7C,D). Furthermore, we found that inhibition of DDX5 expression by FL118 is also associated with the inhibition of mKras (Figure 3F), suggesting that mKras is a potential downstream target of DDX5. To confirm that mKras is a downstream target of DDX5, we performed both silencing of DDX5 and mKras. We found that DDX5 silencing resulted in the complete elimination of mKras (Figure 3G, left panel), while mKras silencing only slightly decreased DDX5 expression (Figure 3G, right panel). These observations indicate that mKras is a downstream target of DDX5, which is of high significance for several reasons. First, the Kras mutation rate is highly prevalent in both CRC and PDAC: the functional mutation rate of Kras in CRC is at a range of 40%–44.7%^76^, ^77^ and in PDAC is at a range of 70%–90%.^78^, ^79^ Second, the mKras is an important target in human PDAC and CRC tumours, especially in PDAC. However, it is very difficult to find a direct and effective inhibitor of mKras. Third, consistent with the finding that mKras is a downstream target of DDX5 (Figure 3G), our recent studies demonstrated that human bladder cancer cells with mKras are more sensitive to FL118 treatment when compared to tumour cells with wild‐type Kras.^80^ Further studies will be required on elucidating the detailed mechanism for DDX5 to control each of DDX5's downstream targets, including mKras.

Additionally, we have observed that while FL118 degrades the DDX5 protein, DDX5 mRNA increases upon FL118 treatment (Figures 2F and 4A). We also observed that while 10 nM FL118 treatment of cancer cells can decrease DDX5, this FL118 concentration for a relatively short‐time treatment (24 h) could slightly increase survivin in some cancer cells (e.g., in SW620 cells but not in Mia Paca‐2, Figure 4B) and mKras cells (Figure 3F). These phenomena are likely a ‘survival compensation action’ through diverse feedback network signalling triggered by FL118‐mediated degradation of DDX5. In other words, through multiple signalling pathways that are directly and indirectly relevant to DDX5 degradation by FL118, cancer cells always try to survive via feedback upon receiving a relatively weaker and/or shorter time (non‐fatal) induced by FL118 treatment. However, sustained loss of DDX5 induced by FL118 would finally send cancer cells into death.

In this report, we used the survivin gene as an example to explore the FL118 potential MOA and signalling pathway by which FL118‐DDX5 regulates the survivin gene transcription. Our studies indicated that FL118 treatment downregulates both the protein and mRNA of survivin and c‐Myc without an inhibitory effect on DDX5 mRNA (Figure 4A,B), suggesting a potential involvement of transcriptional regulation. Consistent with the fact that one of DDX5's functions is to act as a transcription coactivator to interact with TFs for promoting gene transcription, we demonstrated that DDX5 interacts with the TFs of NF‐kB/c‐Rel, c‐Myc and TCF‐4 but not Sp‐1 (Figure 4C). Since there is a DNA‐binding site for c‐Myc in the survivin core promoter region,^57^ we subsequently performed a ChIP assay and demonstrated that c‐Myc binds to the c‐Myc DNA‐binding site in the survivin promoter, and c‐Myc can be abrogated from the c‐Myc DNA‐binding site after FL118 treatment (Figure 4D). Consistent with the previous finding that CDK9 interacts with cyclin T1 (T1) to form a core component of P‐TEFb to interact with c‐Myc^58^, ^59^, ^60^, ^61^ and that DDX5 interacts with CDK9,^62^, ^63^ our studies indicated that DDX5 interacts with both CDK9 and T1 (Figure 4E,F). Taking all of these observations into consideration, along with the previous findings that CDK9/T1 controls extensive crosstalk between TFs and the RNAPII complex for efficient transcription of required mRNA,^64^, ^65^, ^66^, ^81^, ^82^ our studies defined a novel survivin transcriptional regulation pathway through a cascade of FL118‐DDX5‐c‐Myc‐CDK9‐T1‐linked to the RNAPII basic transcriptional machinery in the survivin gene promoter (Figure S6B). Furthermore, given that the promoters of survivin, Mcl‐1, XIAP and cIAP2 share common TF DNA‐binding sites, including the c‐Myc site,^57^ it would be intriguing to determine whether the finding from the use of the survivin gene as an example may or may not represent a general mechanism by which FL118 inhibits multiple antiapoptotic protein genes through FL118 interactions with DDX5. However, FL118 physical and functional control of the DDX5 protein would likely do much more than merely control protein gene transcription. Nevertheless, the findings derived from this report open new doors for further studies of FL118 MOA through its binding to DDX5.

### 3.3 DDX5 target and biomarker role for FL118 molecular precision medicine

The Discovery of DDX5 as the FL118 biochemical target provides a starting point and lays a basis for further extensive elucidation of FL118 MOA in years to come. In addition to the substantial data relevant to the MOA of FL118 through DDX5 (Figures 1, 2, 3, 4) presented in this report, we have also provided extensive in vitro and in vivo data to determine whether DDX5 could be used as an FL118 target and biomarker for predicting PDAC and CRC tumour sensitivity to FL118 treatment (Figures 5, 6, 7, 8, 9). This would be important for future target‐ and biomarker‐driven FL118 clinical trials with PDAC and CRC patients. Meanwhile, we can also take time to further study the MOA for FL118‐DDX5 interaction and signalling regulation as well as in each of DDX5's downstream targets and their associated signalling network during the development of FL118 into clinical application for cancer treatment.

It is our understanding that with an appropriate biomarker, molecular precision medicine can be carried out. We have used both human PDAC and CRC tumour models (Figures 5 and 6) to determine the association of DDX5 expression and tumour sensitivity to FL118 treatment. Our studies indicated that in human PDAC and CRC tumour models, high DDX5 expression is associated with high sensitivity of tumours to FL118 treatment (Figures 5 and 6). Furthermore, we have confirmed the link of the DDX5 status with the FL118 antitumour efficacy using various genetic approaches (Figures 7, 8, 9). Specifically, we demonstrated that the silencing of DDX5 delays PDAC tumour growth (Figure 7A,B), while the OE of DDX5 increases FL118 efficacy to inhibit PDAC cell growth (Figure 7C,D). The high DDX5 that increases FL118 efficacy is largely caused by high amounts of DDX5 targets in cells, making those cells more addicted to DDX5 for survival and thus this would result in FL118 being more efficacious to kill them and/or to inhibit their growth/viability through degrading DDX5. Alternatively, using vector‐free Cas9 enzyme‐DDX5 sgRNA RNP technology to knock out DDX5, we were able to obtain many DDX5 KO cell clones (Figures 8, 9 and S10). We found that these DDX5 KO cell clones are resistant to FL118‐induced cell growth/viability inhibition due to their lack of DDX5 for FL118 to target (Figures 8B,C and 9B,C). Some readers may ask if PDAC cells have no DDX5, these cells should die. Yes, during our selection of DDX5 KO PDAC cell clones, cells that are highly addictive to DDX5 for survival will die after DDX5 KO. The DDX5 KO cell clones that we obtained (Figures 8, 9 and S10) should be those that can survive in a poor to OK range without DDX5. With this being in mind, our obtained DDX5 KO cells would grow slower but survive better upon FL118 treatment (since no DDX5 for FL118 to target) than non‐DDX5 KO control cells (these control cells are more sensitive to FL118 since DDX5 exists for FL118 to target). For the same reason, our studies revealed that DDX5 KO PDAC cells significantly delayed tumour formulation and decreased tumour growth (Figures 8D,E and 9D,E). However, these DDX5 KO cells should be sensitive to FL118 again after re‐expression of DDX5 in their cells. Consistent with this prediction, our studies indicated that re‐expression of DDX5 in the Mia Paca‐2 A6 clone cells made A6 cells re‐sensitive to FL118‐induced cell growth/viability inhibition in comparison with the control A6 cells without re‐expression of DDX5 (Figure S11). These findings strongly support that DDX5 is a bona fide physical, mechanistic and functional target and biomarker to indicate PDAC and CRC tumour sensitivity to FL118 treatment. This is very important because the studies documented in the current literature demonstrated that DDX5 is a superior oncogenic biomarker and target for targeted cancer therapy.^54^

Additionally, during the peer review process, an expert reviewer pointed out that in Figures 7C,D and 8B,C, the effect of FL118 seems to not be concentration‐dependent as it reaches a limit beyond which the increase in concentration (i.e., ≥18.5 nM) does not result in an increase in efficacy. This can also be seen in Figure 7C,D (i.e., ≥ 50 nM). Basically, the explanation of such phenomenon is because FL118 is a targeted drug and uses DDX5 as its therapeutic target, which is quite different from chemo‐cytotoxic drugs, where their efficacy is drug concentration‐dependent and the chemo‐cytotoxic drug dose at its MTD usually exhibits maximal antitumour activity. In other words, when the target protein DDX5 molecules are fully saturated by the FL118 drug molecules in cancer cells, a further increase in the FL118 drug concentration may mainly increase toxicity to normal tissue without further significantly increasing FL118 efficacy. Additionally, consistent with the fact that DDX5 expression and tyrosine phosphorylation are much higher in Mia Paca‐2 cells than those in Panc‐1 cells (Figure S3), FL118 would need higher concentrations to inhibit the non‐DDX5 KO control Mia Paca‐2 cell growth/viability, as shown in Figure 9B,C. Together, these observations are consistent with our conclusion that DDX5 is a bona fide therapeutic target for FL118, and a targeting drug like FL118 may not need to reach its MTD to express its maximum anticancer efficacy.

Furthermore, the in vivo results derived from DDX5 KO PDAC cell tumour formation and growth (Figures 8D,E and 9D,E) provided important mechanistic and treatment outcome predictive information. Specifically, our studies revealed that individual DDX5 KO PDAC cell clones can exhibit different tumour formation and growth abilities (e.g., the A2‐1C clone tumour formation capacity shown in Figure 8D is much lower than the C2‐1 clone tumour formation shown in Figure 8E). In other words, DDX5 KO in individual PDAC cells can result in different cell fates. While this observation is consistent with the fact that cancer cells are heterogeneous, this clearly indicates that the DDX5 target may have differing degrees of importance to different PDAC cells. The DDX5 KO cell clones that were obtained by a limited cell dilution process in 96‐well plates are those that DDX5 KO is not fatal to; in other words, we were unable to obtain DDX5 KO cell clones from cells that will be fetal after DDX5 KO. This knowledge is important to apply to PDAC and CRC treatment to explain why FL118 treatment can only eliminate some of the PDX tumours but not others,^13^ and some eliminated tumours by FL118 treatment can come back (i.e., relapse) later on, while others cannot. Importantly, PDAC PDX tumours showing less sensitivity to FL118 (likely due to low‐level expression of DDX5) can still be eliminated by FL118 in combination with a low level of gemcitabine.^13^ One interesting observation that we would like to point out is that although both PDAC PDX10978 (shown in our previous publication^13^) and PDAC PDX12872 (shown in this study, Figure 5) exhibited relative resistance to FL118 treatment (likely due to low DDX5 expression), their sensitivity to combinational treatment was different. PDX10978 is very sensitive to the FL118 + gemcitabine combinational treatment; a very low‐dose level of FL118 (∼1/10 MTD) in combination with gemcitabine can eliminate PDX10978 tumour.^13^ In contrast,the PDX12872 tumour could be eliminated only at a relatively high FL118 dose level (∼1/2 MTD) in combination with gemcitabine (Figure 5E), suggesting the heterogeneity of individual PDAC tumours. Nevertheless, it is likely that FL118 alone would eliminate some PDAC and CRC tumours, while FL118 in combination with an appropriate chemotherapeutic agent would eliminate some other PDAC and CRC tumours with low levels of DDX5 expression. With this strategy, we could maximise the clinical outcome for most (if not all) PDAC and CRC patients.

Additionally, we also observed that the same DDX5 KO clone can exhibit different tumour growth rates. For example, the growth of the C2‐1 clone tumour in different individual mice exhibited different tumour growth rates as shown in Figure 8E. This suggests that different individual mice with the same tumour background could have different tumour formation and growth acceptability (i.e., tumour‐host effects), which is understandable and matches the human cancer situation seen in the clinic.

Together, our studies have demonstrated that DDX5 is a bona fide FL118 target and biomarker for predicting PDAC and CRC tumour sensitivity to FL118 treatment. However, to maximise the future clinical outcome of FL118 application and to understand the detailed FL118 MOA, further studies are required to assess targeting DDX5 by FL118 alone (most patients would be enough) or in combination with appropriate chemotherapeutic agents (for some patients who have a low‐level expression of DDX5 in their PDAC or CRC tumours). Thus, most (if not all) PDAC and CRC patients can be qualified for an effective treatment using FL118, alone or in combination with a low level of conventional chemotherapeutic agents.

### 3.4 Final remarks

**FIGURE 10:**
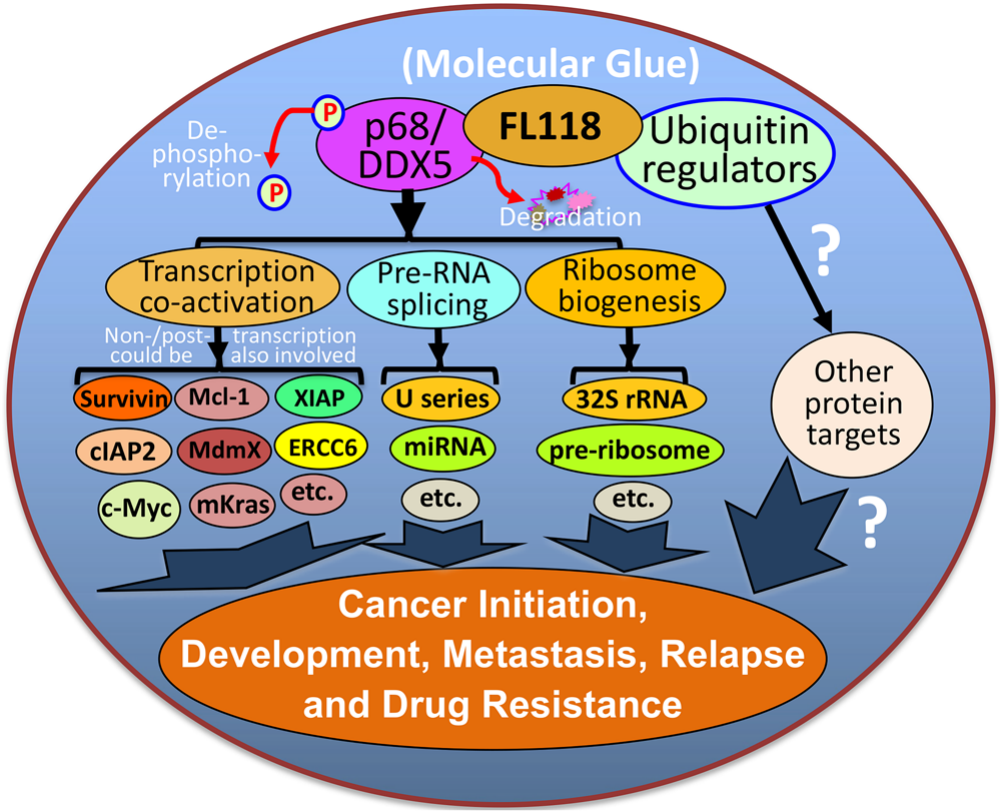
Diagram of the FL118 mechanism of action to elucidate why FL118 possesses extraordinary antitumour efficacy. DDX5 (also called p68) is a multifunctional master regulator involved in (1) co‐activation of transcription of many oncogenes through the direct interactions of different transcription factors (e.g., c‐Myc) in the oncogenic gene promoters, (2) regulation of miRNA and pre‐RNA splicing (e.g., U1, U2, U3, … snRNP), and (3) ribosome biogenesis (e.g., 32S rRNA, pre‐ribosome). The novel small molecule drug FL118 binds to and functionally dephosphorylates and degrades the DDX5 protein (without decreasing DDX5 mRNA) through the proteasome degradation pathway, suggesting that FL118 could glue both DDX5 and ubiquitin‐involved protein stability/degradation regulators (i.e., FL118 acts as a “molecular glue degrader”). All the DDX5 downstream protein targets are known to be involved in cancer initiation, development, metastasis, recurrence and treatment resistance. Therefore, indirectly blocking DDX5 downstream targets through direct dephosphorylation and degradation of DDX5 by FL118 could result in FL118 high antitumour efficacy as demonstrated in this study, which used human CRC and PDAC cell and tumour models.

Given the documented functions of DDX5 in oncogenic gene transcription,^31^, ^33^, ^35^, ^37^, ^53^, ^83^ various types of RNA splicing/regulation^38^, ^39^, ^40^, ^41^, ^84^, ^85^ and ribosome biogenesis,^42^, ^43^ as well as based on our breakthrough findings presented in this study, we provide an outline (Figure 10) to justify the high antitumour efficacy of FL118 acting as a potential molecular glue degrader through its distinct MOA plus other possible FL118 biochemical targets via ‘molecular glueing’ for future studies in the cancer community. Readers can refer to the recent review article for more information on the molecular glue topic.^75^ On a further note, most (if not all) molecular glue compounds could bind to more than one protein target. However, when a molecular glue compound becomes a drug, as long as the binding has good specificity, binding to more than one protein target will not be an issue to induce toxicity. For example, the very successful drug Revlimid (lenalidomide, which is a small molecule molecular glue compound) binds to at least 13 functional proteins.^86^ However, toxicity was not an issue for Revlimid in its approval by the FDA for the treatment of multiple myeloma. In this regard, a molecular glue compound having multiple targets may be an advantage, instead of a weakness, for the compound to exhibit high antitumour activity.

## 4 MATERIALS AND METHODS

In this study, we used both in vitro and in vivo PDAC and/or CRC cell and tumour models with genetic and/or pharmacological approaches to silence, overexpress and knock out the relevant target genes/proteins to determine their molecular and mechanistic relationship in the signalling network as well as the focused anticancer small molecule FL118 efficacy to treat PDAC and CRC tumours.

### 4.1 Cell lines, cell culture and reagents

The human embryonic kidney 293T (HEK293T) cell line, human PDAC cell lines (Panc1, MiaPaca2) and human CRC cell lines (HCT‐8, SW620, SW480, SW837, SW948, NCI‐H716, SNU‐C2B, Colo205) were originally purchased from ATCC. All of these cell lines were maintained in either DMEM or RPMI 1640 medium supplied with 10% fetal bovine serum (FBS, Atlanta Biologicals), penicillin (100 units/ml) and streptomycin (0.1 μg/ml; Thermo Fisher Scientific/Invitrogen). Cells were routinely sub‐cultured twice a week and maintained in a humidified incubator with 5% CO_2_ at 37°C. Monoclonal anti‐tubulin antibody, polyclonal anti‐actin antibody and goat peroxidase‐conjugated anti‐rabbit IgG antibody were purchased from Sigma or Invitrogen/Thermo Fisher Scientific. Antibodies against survivin (FL‐142), c‐Rel/NF‐kB, Sp‐1, normal IgG and GAPDH were from Santa Cruz. DDX5 antibodies from R&D Systems and Sino Biological. Antibodies against c‐Myc, Mcl‐1 and cyclin D1 were purchased from Proteintech. Antibodies against CDK9 and cyclin T1 were purchased from Santa Cruz. Top1 antibodies were purchased from TopoGen (TG2012‐4, Lot12FB04) and BD Biosciences (X‐21, RUO). Antibodies against Kras, mKras, cIAP2, XIAP, Mcl‐1, TCF‐4, c‐Myc, survivin, Sp‐1, caspase‐3 (cleaved and full length) and PARP (cleaved and full length) were purchased from Cell Signaling. MTT and leupeptin were purchased from USB/Affymetrix. BSA was purchased from EMD. Topotecan, SN‐38 and MG132 were purchased from Selleck Chemicals. pCMV6‐Entry‐Myc‐DDK‐DDX5 and pCMV6‐Entry‐Myc‐DDK‐Top1 expression vectors were purchased from OriGene. Ubiquitin‐specific antibody was purchased from UBPBio.

### 4.2 Affinity column purification of FL118‐binding proteins

The purification was based on the use of a CarboxyLink Immobilisation kit with UltraLink Support (Part# 53154) from Thermo Scientific. The process includes many experimental steps: (1) FL118 ligand coupling to the resin slurry: (a) equilibrate two columns of DADPA UltraLink Support and the bottle of Wash Buffer to room temperature, and then remove caps from the column and set the two warmed columns onto a homemade rack to allow the storage solution to drain from the column; (b) equilibrate column by adding 2 ml of coupling buffer (CB) and allowing it to flow through the resin bed and drain from the column. Then, gradually equilibrate the resin with 3 ml (column volume is 2 ml) of each of the following solutions with gradual increased DMSO from 10% to 80% before adding FL118 in 80% DMSO:20% CB. Discard flow‐through from the collection test tube; (c) add 5‐ml control solution (4.5‐ml DMSO + 0.5‐ml CB) to Column 1 and in parallel, add 5‐ml FL118 solution [2.5‐ml FL118 (1‐mg/ml in DMSO) + 2‐ml DMSO+ 0.5‐ml CB] to Column 2; (d) resuspend the resin in the control or FL118 solution by gentle reverse up and down; (e) transfer the resin slurry to a 15‐ml conical tube labelled with Columns 1 and 2; (f) add 200‐μl PharmaLink Coupling Reagent (37% formaldehyde solution) in each 15‐ml tube: 200 μl per 2–4‐ml FL118 binding solution); (g) cap the tube and incubate the control Column 1 slurry and Column 2 FL118 slurry at 54°C in swirling for ∼ 24 h; (h) then add 1000‐μl CB plus 50‐μl formaldehyde to each column swirling at 55°C for 48 h. (2) Affinity purification column setup: (a) transfer the control resin slurry and the FL118‐coupled resin slurry into two columns; (b) allow the resin to settle and then open the bottom cap to allow the solution to flow out to drain all the reaction solution; (c) wash the column with 2‐ml CB and then with 2‐ml ultrapure water/DMSO solution (75:25). Then wash with 1.5‐ml 100% DMSO to help remove non‐reacted FL118. (d) continuously wash the column with 5‐ml CB on each column. Then wash with 4‐ml tris washing buffer (0.1 M Tris, pH 8.0) and then continue to wash the column with 9 × 4 ml tris washing buffer (0.1 M Tris, pH 8.0) to quench the active sites that were not sealed by FL118. On the final washing, drained to ∼2 ml, recap column and put columns at oven at 32–37°C for 45 min to facilitate quenching the active sites that were not sealed by FL118. Then, an additional 3 × 4 ml tris washing buffer (0.1 M Tris, pH 8.0). (3) Affinity column purification of FL118‐binding proteins: Cancer cell lysates (2 × 5 mg) were passed through an affinity column and control column in parallel. After extensive washing with washing buffer, the proteins binding to the column were eluted with 8‐M urea buffer. (4) Protein display in gel: After the eluted protein solution de‐urea and concentration into 20–30 μl through a 3K OMEGA NANOSEP 1.5 ml tube device (PALL Life Sciences), the entire resulting protein mixtures from the control column and the FL118 affinity column in parallel were displayed on a 5%–20% gradient SDS PAGE gel, and the displayed unknown protein band was isolated for protein identification.

### 4.3 MS identification of the unknown protein that binds to FL118

After the unknown protein bend in the FL118 column and the corresponding gel area from the control column were isolated from the gel display, in‐gel digestion was performed by adding trypsin (sequencing grade, Promega, 10 ng/μl in 10% (v/v) acetonitrile, 40 mM ammonium bicarbonate) to dehydrate gel slices and incubated at 37°C for 16 h. After digestion, the tryptic products were extracted twice and dried; the dried peptides were then reconstituted in 10 μl of 2% (v/v) formic acid for analysis with liquid chromatography tandem MS (LC‐ESI‐MS/MS). LC‐ESI‐MS/MS analysis of peptides was performed using a nano‐ACQUITY UPLC system (Waters Corporation) coupled through a nano‐spray ionisation source to a quadrupole‐time‐of‐flight (Q‐ToF) Premier mass spectrometer (Micromass). The reconstituted tryptic digests in formic acid were loaded and eluted with a gradient of 99% solvent A (0.1% (v/v) formic acid)/1% solvent B (0.1% (v/v) formic acid in acetonitrile) to 10% solvent A/90% solvent B at 0.8 μl per min. For fragmentation analysis, the Q‐ToF mass spectrometer was programmed to select ions with a mass/charge in the range of 300–1500 Da and ions with +2 to +5 charges. All LC‐MS/MS spectra were transformed to the Micromass Pick List (PKL) file formats using the ProteinLynx Global SERVER and the default parameters of the MaxEnt3 algorithm. The PKL file was used to search against humans on a local MASCOT server using the following search parameters: (1) trypsin as the proteolytic enzyme with two possible missed cleavages; (2) carbamidomethylation of cysteine as a fixed modification; (3) oxidation of methionine as a variable modification; (4) an allowable mass error of 100 ppm for peptides and 0.1 Da for fragment ions; (5) the peptide charge set to 2+ and 3+; and (6) the instrument option set to ESI‐QUAD‐TOF. The criteria used to identify the proteins included ion scores equal to or greater than the threshold significant ion score (*p *< 0.05) and similarity between the observed protein molecular mass from gel electrophoresis and the calculated molecular mass provided in the database. The eluate from a negative control column (no FL118) contained only keratins as expected.

### 4.4 Purification of Flag‐DDX5 and Flag‐Top1 protein

The Flag‐DDX5 and Flag‐Top1 proteins were purified from the protein expression vector‐transfected HEK293T cells by using the FLAG M Purification Kit for mammalian expression systems (CELLMM2‐1KT, Sigma). Briefly, sub‐confluent populations of HEK293T cells were transfected with Flag‐DDX5 vectors (RC200371, OriGene) and Flag‐Top1 vectors (RC215658, OriGene) using Lipofectamine 2000 (Invitrogen) as per the manufacturer's instructions. After 72 h of transfection, cells were lysed in Cell Lytic M lysis reagent (Sigma Cat # C2978) containing a protease inhibitor cocktail. Supernatants were collected by centrifugation at 13 000 rpm for 10 min at 4°C. The Flag fusion proteins from the cell lysates were purified with the ANTI‐FLAG M2 affinity gel, which is a highly specific monoclonal antibody covalently attached to agarose resin. For this purification step, the required amount of ANTI‐FLAG M2 resin was transferred to a fresh microcentrifuge tube and washed twice with 0.5 ml of 1X wash buffer (50 mM Tris‐HCl, 150 mM NaCl, pH 7.4). Of note, 10X wash buffers were provided in the kit. The washed resin was then added to the protein extract in a microcentrifuge tube and agitated in a roller shaker overnight. After this binding step, the resin was collected by centrifugation and washed three times with 1X wash buffer to remove non‐specific proteins. The bound Flag‐tagged proteins were eluted from the resin by competitive elution with the 3X Flag peptide (catalogue number F4799) in 1× wash buffer.

### 4.5 Labelling of FL118 with ^3^H

Preparation of itritum (^3^H)‐labelled FL118 was performed by Moravek Biochemicals through service. (1) Generation of ^3^H‐labelled FL118 with a specificity of 5.6 Ci/mmol: The 3H labelling of FL118 using ^3^H/^1^H exchange approaches. Specifically, a 50‐ml round bottom flask was charged with 1‐mg FL118, 100‐mg PdBaSO4 5% and 60 Ci T2 gas. The flask was then immersed in a silicone oil bath at 180– 190°C for 6 h. The ^3^H gas was removed. The reaction mixture was dissolved in 1 ml of DMSO and backexchanged 10X with 50% ethanol. Injected directly to a CapCeLL PAK C‐18 column (4.6 × 250 mm), mobile phase 30% CH3CN, 0.1% TFA, flow 1 ml/min, U.V. = 200 nm, r.t. = 40 min. After purification, the total activity was 5 mCi (solid) with a specificity of 5.6 Ci/mmol at a radiochemical purity of ≥ 97%. (2) Generation of ^3^H‐labelled FL118 with a specificity of 16.5 Ci/mmol: The 3H labelling of FL118 using ^3^H/^1^H exchange approaches. Specifically, a 50‐ml round bottom flask was charged with 1‐mg FL118, 120 mg PdBaSO4 5% solution and 90 Ci T2 gas. The flask was then immersed in a silicone oil bath at 190°C for 24 h. The ^3^H gas was removed. The reaction mixture was dissolved in 1‐ml of DMSO and backexchanged 10X with 50% ethanol and injected directly into a CapCeLL Pak C‐18 column (4.6 × 250 mm), mobile phase 30% CH3CN, 0.1% TFA, flow 6 ml/min, U.V. = 200 nm, r.t. = 40 min. After purification, the total activity was 5 mCi (solid) with a specificity of 16.5 Ci/mmol at a radiochemical purity of ≥ 81%.

### 4.6 Determination of FL118‐DDX5 and FL118‐Top1 binding using a Nanosep device

The binding of FL118 to DDX5 versus Top1 was alternatively determined using the low protein‐binding polypropylene Omega membrane Nanosep 3K Centrifugal Devices (PALL, Life Sciences), which can let molecules with a molecular weight size of less than 3 kD pass while retaining 100% of molecules larger than 10 kD. Briefly, ^3^H‐labelled hot FL118 (^3^H‐FL118) was mixed with non‐labelled cold FL118 at a ratio of 1:10 to make an FL118 concentration of 10 μM in 1x phosphate‐buffered saline (PBS, pH 7.4) containing 8% DMSO (designated as hot FL118 solution). Then, 5–10 μg of the FLAG M Purification Kit (Signa)‐purified Flag‐DDX5 and Flag‐Top1 proteins in a volume of 20–50 μl was transferred into the sample reservoir of a 1.5 ml Nanosep 3K Centrifugal Device. In parallel, a negative control Nanosep 3K centrifugal devices containing 10‐μg BSA in a 50 μl volume were also set up. Then, the three prepared Nanosep 3K devices were centrifuged at 10K rpm for 10–15 min to eliminate the solution and retain the proteins in the sample reservoir. Next, the proteins in the reservoirs of the three Nanosep 3K devices were resuspended with 100–200 μl of hot FL118 solution for FL118‐protein binding at room temperature for 30 min. Then, the free FL118 in the solution was removed by a low speed of centrifugation (2000–4000 rpm) of the three Nanosep 3K devices for 15–30 min until there was no solution in the sample reservoir. Last, all three of the sample reservoir devices were transferred into a scintillation vial containing enough scintillation solution to cover the entire sample reservoir devices for ^3^H counting on the LS 6500 Scintillation System (Backman Couler).

### 4.7 Determination of FL118‐DDX5 and FL118‐Top1 binding affinity

The binding affinity of FL118 with DDX5 versus Top1 was determined using the ITC, a state‐of‐the‐art technology for measuring small molecule‐protein interactions to define the drug‐protein affinity (K_D_). Briefly, (1) FL118 solution (≥160 μL/sample, required volume for testing on the MicroCal‐Malvern Auto‐ITC200 equipment) containing 100 μM FL118 in 1x PBS (pH 7.4) and 8% DMSO from 2 mM FL118 stock in DMSO (the same solution was also prepared for topotecan) was prepared. The minimal DMSO concentration in the solution was determined in advance, and the 8% DMSO was the optimal concentration to use in order to balance the lowest concentration while there was no FL118 precipitation (Table S2); (2) preparation of Flag‐DDX5 protein solution and Flag‐Top1 protein solution (≥360 μl/sample), containing 10 μM Flag‐DDX5 protein or 10 μM Flag‐Top1 protein in 1x PBS (pH 7.4) and 8% DMSO from protein stocks in 1x PBS (the same solution was also prepared for BSA); (3) preparation of washing/diluting solution (≥2.5 ml/sample) containing 1x PBS (pH 7.4) and 8% DMSO; and (4) ITC assay was performed using the auto‐iTC200 instrument (MicroCal, GE). A protein solution (Flag‐DDX5, Flag‐Top1 or BSA) with a defined concentration (10 μM) in a volume of 360 μl in 1x PBS (pH 7.4) containing 8% DMSO was loaded into the calorimetre cell as the macromolecule (M). Then, a small compound ligand (FL118 or topotecan) at a concentration of 10x protein concentration (100 μM) in the same buffer containing 8% DMSO was directly injected into the M cell at a volume of 2 μl per injection every 3 min for equilibrium in a total of 20 injections in 1 h at 25°C. At each injection, the program calculates [M]t and [X]t (total protein and total ligand at that injection). The binding constant (K_D_), binding stoichiometry (n) and thermodynamic parameters (ΔH and ΔS) were determined by fitting the titration curve to a one‐site binding mode using Origin software provided by the manufacturer.

### 4.8 Preparation of FL118 for in vitro and in vivo application

FL118 was synthesised in‐house with a purity ≥ 99%. For in vitro cell culture studies, FL118 was initially dissolved in DMSO at a concentration of 1 mM as the stock solution for further dilution to various concentrations in the appropriate medium for the experiment. For in vivo studies, FL118 used a basic formulation recipe, which contained FL118 (0.1 ‐ 0.5 mg/ml), DMSO (0%–5%, v/v) and hydroxypropyl‐β‐cyclodextrin (0.1%–0.5%, w/v) in saline containing 2.5% propylene glycol (PG) and 2.5% PEG400. The formulation process was described in detail in the published patents (USA patent US 7,569,221 B2, 2009; PCT/US2011/058558, USA2011; and PCT/US2015/022095, USA2015). The vehicle solution contained the corresponding concentrations of DMSO (0%–5%) and hydroxypropyl‐β‐cyclodextrin (0.1%–0.5%) in saline containing 2.5% PG and 2.5% PEG400 without FL118. FL118 in the formulated suspension for in vivo oral administration is highly stable for more than 24 months and has no observable changes in its antitumour efficacy in comparison with the freshly prepared compound when tested in human tumour animal models.

### 4.9 IP assays

Pertinent to this study, for IP with DDX5 or normal IgG antibodies, SW620 cells were treated with and without FL118 (100 nM, 500 nM) for 6 and 24 h, respectively. Up to 10^7^ cells for each time point/test were harvested with a scraper in cold PBS buffers and collected by centrifugation at 1000 g x 5 min and washed with cold PBS once. Cell pellets were lysed in 1 ml cold RIPA lysis buffer containing proteinase inhibitors (refer to western blots). The cell lysates were precleared by centrifugation at 14 000 rpm for 15 min. The resulting supernatants were transferred to a new 1.5 ml tube and incubated with up to 3 μg either DDX5 antibodies or normal IgG antibodies for 1 h at 4°C, followed by incubation with 20–40 μl 50% protein A/G‐agarose slurry overnight at 4°C on a rocker platform. After centrifugation at 1000 g for 5 min at 4°C, the pellet was washed with RIPA buffer or PBS for two to four times and collected by centrifugation as above. The pellets were then resuspended in 20–40 μl of 1x sample buffer, boiled for 2–4 min and stored on ice for western blot analyses.

### 4.10 Western blot (immunoblotting) analyses

For western blot pertinent to this study, cancer cells that were treated with and without FL118 (in some experiments in the presence of MG132) were lysed in RIPA buffer containing 150 mM NaCl, 1.0% IGEPAL CA‐630, 0.5% sodium deoxycholate, 0.1% SDS and 50 mM Tris, pH 8.0. Thirty to fifty micrograms of total protein from each sample was heated at 95°C for 5 min after mixing with equal volumes of 2X SDS loading buffer. Samples were separated on 10%–15% SDS‐polyacrylamide gel electrophoresis (SDS‐PAGE) gels and electro‐transferred to Pure Nitrocellulose Membranes with either 0.45 or 0.2 μm (Bio‐Rad) based on the tested protein size. The membrane was then blocked in 5% skim milk in TBS‐T buffer (20 mM Tris/HCl pH 7.5, 0.137 M NaCl and 0.1% Tween 20) at room temperature for 2–3 h. Next, the membrane was incubated with different primary antibodies in TBS‐T containing 5% BSA overnight at 4°C in the range of dilutions from 1:500 to 1:2000 based on the antibody dilution recommended by antibody firms. After washing with TBS‐T, the membrane was incubated in TBS‐T buffer containing 5% skim milk and the corresponding secondary antibody (1:5000) for 45–60 min at room temperature with shaking. Proteins of interest were detected using Western Lightning ECL‐Plus (Perkin Elmer) and visualised by various times (3–120 s) of exposure. Actin, tubulin and/or GAPDH were detected as the internal control to normalise the total protein loading for each sample.

### 4.11 Real‐time quantitative (q) RT‐PCR

Real‐time reverse transcription (RT)‐PCR methods were described previously.^9,5^ Pertinent to this study, total RNA was extracted from cancer cells using TRI REAGENT RT (Molecular Research Center), and 5 μg per sample was converted to cDNA with anchored oligo (dT) primers using a RevertAid First Strand cDNA Synthesis Kit (Thermo Fisher Scientific, Catalog No. K1622) following the manufacturer's instructions. Individual RT reactions of 20 μl were then diluted to 200 μl with sterile H_2_O. Five microlitres of diluted RT reaction was used for real‐time qPCR using Maxima SYBR Green/ROX qPCR Master Mix (2X, Thermo Fisher Scientific, Catalog No. K0223). The primers used in qPCR were as follows: 5′‐GGC GCA CAG CAC AAG AGG‐3′ (DDX5 qPCR‐F, forward) and 5′‐ ATG GCA GGA AGC AAA TAA GAC AA‐3′( DDX5 qPCR‐R, reverse); 5′‐CTT CTG CTT CAA GGA GCT GGA AG‐3′ (hsv5p2, survivin, forward) and 5′‐GCA CTT TCT TCG CAG TTT CCT C‐3′ (hvs3p2, survivin, reverse); 5′‐CCA CAG CAA ACC TCC TCA CAG‐3′ (myc5, c‐Myc, forward) and 5′‐GCA GGA TAG TCC TTC CGA GTG‐3′ (myc3, c‐Myc, reverse); 5′‐GTC TCC TCT GAC TTC AAC AGC G‐3′ (hGAPDH‐5, forward) and 5′‐ACC ACC CTG TTG CTG TAG CCA A‐ 3′ (hGAPDH‐3, reverse). GAPDH was used as an internal control. Triplicate qPCR reactions were performed for individual samples. The qPCR condition is 50°C for 2 min and 95°C for 10 min as a pre‐denature step, followed by 40 cycles at 95°C for 15 s and 60°C for 1 min in a thin‐wall 96‐well PCR plate. The data were analysed using the Applied Biosystems 7300 Real‐Time PCR System and normalised to GAPDH.

### 4.12 Preparation of lentiviral particles containing either DDX5 shRNA or cDNA gene

For the lentiviral infection particles preparation pertinent to this study, DDX5 shRNA1 and DDX5 shRNAs2 (Figure S5) in the pGIPZ lentiviral vectors (with puromycin for selection and TurboGFP as visual marking of shRNA expressing cells) in the bacterial stock from the GIPZ lentiviral shRNAmir library (Roswell Park Comprehensive Cancer Center [Roswell Park] shRNA Resource in collaboration with Open Biosystems) were prepared using midi prep kits. The HEK293T packaging cells at 80% confluence were incubated for 24 h at 37°C and 5% CO_2_ and transfected by gently replacing the cell medium with 500 μl DNA/Lipofectamine 2000 complex with gentle swirling. Three millilitres of DMEM with 10% FBS and 1% Pen/Strep were added after a few minutes and then placed in a 5% CO_2_ incubator for 16 h at 37°C. The 500 μl DNA/Lipofectamine 2000 complex was prepared as follows: 250 μl DMEM containing 2.5 μg pGIPZ shRNA, 2.5 μg psPAX2 (or pCMV‐dR8.74), 1.0 μg pMD2. G in one tube was mixed with 250 μl DMEM containing 9–12 μl Lipofectamine 2000 and kept at room temperature for 20 min. The medium in the transfected HEK297T cells in the dish was replaced with new media on the next day, and the dish was incubated in a 5% CO_2_ incubator for an additional 24 h at 37°C. Virus‐containing supernatant was harvested and filtered through a 0.45‐μm cellulose acetate (low protein binding) syringe filter, and the virus was stored at 4°C until use. The TurboGFP expression was checked before collection of the virus in the supernatant. The transfected 293T cells in the dish were added to another 3 ml of media and incubated in a CO_2_ incubator at 37°C overnight. The supernatant collected as above was then combined with the second batch supernatant as the lentiviral particle stock stored at 4°C for the experiments.

Additionally, we amplified the DDX5 cDNA by PCR using pCMV6‐Entry‐DDX5 (Origene, with a Flag tag) as a template, and the PCR products were cloned into the pLenti6/V5‐D‐TOPO vector (Thermo Fisher). Of note, the pLenti6⁄V5 Directional TOPO Cloning Kit contains the TOPO‐adapted ViraPower lentiviral expression vector, pLenti⁄V5‐D‐TOPO for quick PCR‐based cloning and high‐level expression of a target gene in dividing and non‐dividing mammalian cells. The vector has the CMV promoter for driving high‐level, constitutive expression of the target gene and the blasticidin selection marker for stable selection in mammalian cells. The lentiviral particle preparation was the same as above for DDX5 shRNA lentiviral preparation.

### 4.13 Target cell infection with lentiviral particles

For infection of target cells with lentiviral stock, cancer cells grown to sub‐confluence in 6‐well plates were infected with 1 ml of lentiviral stock prepared as above in the presence of 4 μg/ml polybrene (infection stimulator). To increase cell infection rates, the plate was spun at 1800 rpm for 45 min at room temperature on a microtiter rotor. The infected cells in the plate were then incubated in a CO_2_ incubator at 37°C for 3–6 h, an additional 1 ml of complete media was added, and the cells were incubated overnight in the CO_2_ incubator at 37°C. Cells in individual wells were then diluted five times (one plate to five plates) and incubated for 24 h, followed by selection with puromycin (2 μg/ml) for 3–7 days. The resultant puromycin‐selected cells were directly used for the experiments or were further used for obtaining single‐cell clones.

### 4.14 MTT assay for determining cancer cell growth/viability

The MTT assay was described previously.^13^ Pertinent to this study, CRC or PDAC cell growth after genetic OE of DDX5 and/or FL118 treatment at different concentrations was determined by MTT (3‐(4,5‐dimethylthiazol‐2‐yl)‐2,5‐diphenyltetrazolium bromide) cell growth/viability assay. Briefly, ∼2500 viable cells with or without genetic manipulation were plated in each well in 96‐well plates. After being incubated overnight in a 5% CO_2_ incubator at 37°C, cells were treated with and without FL118 at different concentrations and continuously incubated for 72 h. MTT, a colorimetric substrate, was then added to a final concentration of 0.4 mg/ml to each well. Cells in 96‐well plates were further incubated in a 5% CO_2_ incubator at 37°C for 4 h, and then the medium was aspirated. The MTT metabolic product formazan was solubilised by adding 200 μl of DMSO to each well. Absorbance in the relevant wells was measured at 570 nm using an Ultra Microplate Reader (Bio‐Tek Instruments).

### 4.15 ChIP assay

For chromatin immunoprecipitation (ChIP) assay experiments pertinent to this study, the SimpleChIP Enzymatic ChIP Kit (Magnetic Beads) (#9003S, Cell Signaling Technology) was used according to the manufacturer's protocol. Briefly, sub‐confluent SW620 cells grown in 3 × 15 cm culture dishes containing 20 ml RPMI‐1640 medium (10% FBS) in each dish were treated with vehicle (DMSO, control) and FL118 (100 nM) for 6 and 24 h, respectively. Then, the cells were crosslinked with 1% formaldehyde by adding 540 μl of 37% formaldehyde to the medium and incubated at RT for 10 min; next, the reaction was quenched by adding 2 ml of 10X glycine, which was provided in the kit. After the cells in the dish were washed with ice‐cold PBS twice, the cells were lysed in 600 μl of SimpleChIP Enzymatic Cell Lysis Buffer B and digested with 3 μl of micrococcal nuclease at 37°C for 20 min. Then, the pellet was collected by centrifugation at 13 000 rpm at 4°C for 1 min. The pellet was then resuspended in 700 μl of ChIP buffer and sonicated for 2 min using Sonicator 3000 (Misonix), and the supernatant (cross‐linked chromatin preparation) was collected by centrifugation at 10 000 rpm at 4°C for 10 min, and then 100 μl for each ChIP in each condition. ChIP in each condition (control, 6 h, 24 h) was performed for c‐Myc antibody (#13987S, Cell Signaling Technology) using 10 μg as recommended by the company, for Histone H3 (D2B12) XP Rabbit mAb (#4620, Cell Signaling Technology) using 2 μg as recommended, and for Normal Rabbit IgG (#2729, Cell Signaling Technology) using 2 μg as recommended. Each of the ChIP complexes was collected using Protein G Magnetic Beads. After the ChIP complexes were eluted in 150 μl ChIP elution buffer provided in the kit from the Protein G Magnetic Beads, we added 6 μl 5 M NaCl and 2 μl proteinase K (#10012) to reverse cross‐linking and release DNA; then, the released DNA was purified using spin columns provided in the kit. The resultant DNA was amplified using the survivin promoter c‐Myc binding site‐relevant primer set or the survivin gene intron primer set by PCR using AccuPrime Taq DNA polymerase. PCR products were visualised with ethidium bromide staining after the experimental samples were separated on 2% agarose gels. The following primer sets were used: The survivin promoter c‐Myc binding site relevant primer set was 5′‐GAG ACA AGG TTT CAC CGT GAT‐3′ (survivin promoter c‐Myc region primer, forward) and 5′‐GAG CGC ACG CCC TCT TA‐3′ (survivin promoter c‐Myc region primer, reverse). The survivin intron‐relevant primer set was 5′‐ATG CCA TAT TCT TTT CTC ACC TT‐3′ (negative control intron primer, forward) and 5′‐GGA CCC CCT AGC TCA CAC TCT CA‐3′ (negative control intron primer, reverse).

### 4.16 Vector‐free CRISPR‐Cas9 technology to knock out DDX5 in PDAC cells

The CRISPR–Cas9 technique was performed to knock out the DDX5 gene in Panc‐1 PDAC cells. To simplify the DDX5 KO process, instead of using expression vectors in the classical approach, we directly used the DDX5 sgRNA‐Cas9 enzyme protein RNP complex through electroporation transfection for KO of the DDX5 gene in pancreatic cancer cells. Specifically, we first ordered the DDX5‐Alt‐R CRISPR‐Cas9 crRNA (a part of sgRNA), Alt‐R CRISPR‐Cas9 tracrRNA (another part of the sgRNA) with ATTO 550 (Cat# 1075928), Alt‐R S.p. Cas9 Nuclease V3 (Cat# 1081058), Alt‐R Genome Editing Detection Kit (Cat# 1075932), IDTE pH 7.5 (1X TE solution; Cat# 11‐01‐02‐02) and Alt‐R Cas9 Electroporation Enhancer (Cat# 1075915) from Integrated DNA Technologies; we also made orders of QuickExtract DNA extract solution (Cat# QE0905T) from Lucigene and NeonTransfection System 10 μl kit (Cat# MPK1096) from Thermo Fisher Scientific. Then, DDX5 sgRNAs were formed into the RNP complex at room temperature in vitro with the Cas9 enzyme in a process with defined conditions using the ordered reagents. PDAC cells were harvested from 10‐cm tissue culture dishes, washed with 5 ml PBS and collected by centrifugation to make 5 × 10^5^ cells in a 9 μl resuspension buffer R (from NeonTransfection System 10 μl kit). Then, 9 μl of cells + 1 μl of RNP complex from above + 2 μl of Alt‐R Cas9 Electroporation Enhancer (18 μM) were mixed, and 10 μl of this mixture was used for electroporation at 1600 V, 10 ms pulse width and 3 pulses. After electroporation, the cells were transferred to a 6‐well plate containing 3 ml of complete DMEM cell culture medium and incubated at 37°C with 5% CO_2_ for 72 h. Next, the positive transfection was validated through the observation of presence of fluorescence in the cells. Specifically, the Alt‐R CRISPR‐Cas9 tracrRNA labelled with ATTO 550 fluorescence allows detection and intracellular visualisation of molecular components via fluorescence microscopy. The fluorescence signal was detected after 48 h of electroporation under fluorescence microscopy to verify the positive transfection. After 72 h of electroporation, cells were collected from the cell culture dish. Half of the cells were used for Alt‐R genome editing detection, and the other half was used for single‐cell cloning.

To isolate single DDX5 KO cell clones from the electroporated Panc‐1‐cell pool and the electroporated Mia Paca‐2 cell pool, the cell pools were processed with a series of dilutions in wells containing 200 μl DMEM complete medium in 96‐well plates to the degree that would make many single wells contain only a single cell. After observation of single cells forming colonies in a single well under the microscope, the cells were transferred to a 6‐well plate containing 3 ml of DMEM complete medium for further culture. Cells were harvested at 80% confluence, and half of the cells was used for the detection of DDX5 OK by western blotting and the other half cells were left for further growth in the 6‐well plate. Western blot‐positive cell clones were further amplified, and some of them were deposited in liquid nitrogen, while others were used for functional analysis.

### 4.17 Methods to detect DDX5 KO cells in the electroporation‐derived live‐cell pool before isolation of single‐cell clones

To detect Alt‐R genome editing, the cells were washed with 100 μl of PBS, lysed with 50 μl of QuickExtract DNA extract solution and heated at 65°C for 10 min, followed by 98°C for 5 min. The genomic DNA was diluted by adding 100 μl of nuclease‐free water, and 4 μl was used for each PCR using the PCR primer pairs that were designed to amplify the DDX5 target site and adjacent sequence: Primer pair A (PCR products length 832 bp): Cas9_DDX5_AA_AB‐F: 5′‐GAT GGC CAG TTG CTC TAA GTG‐3′ and Cas9_DDX5_AA_AB‐R: 5′‐TCA AAG CCC ATA TCA AGC ATT CT‐3′. Primer pair B (PCR products length 749 bp): Cas9_DDX5_AA_AC‐F: 5′‐TCC AAA ACG GCC ATA TGA GTA ACA‐3′ and Cas9_DDX5_AA_AC‐R: 5′‐CCC AAA GCC ACC TAT ATC CAA AAG‐3′. After PCR, the PCR products were used to form heteroduplexes for T7 endonuclease (T7EI) digestion: 10 μl of PCR products, 2 μl of T7EI reaction buffer and 6 μl of nuclease‐free water (18 μl total) were incubated at 95°C for 10 min, cooled to 85°C for 1 min and then cooled to 25°C to form heteroduplexes. For T7EI digestion, 2 μl of T7EI (1 U/μl) was added to 18 μl of heteroduplexes and incubated at 37°C for 60 min, and then digested products were visualised by running agarose gel.

### 4.18 Human tumour specimens, human PDX tumour mouse model and treatment

Clinical CRC and PDAC tumour tissues (fresh or frozen) were originally obtained from Roswell Park Hospital Clinic by using deidentified patient clinical tumour tissues under IRB protocols that were determined to constitute non‐human subject research, including protocol numbers BDR‐063015 (for CRC tumour tissues/specimens) and BDR‐111819 (for PDAC tumour tissues/specimens). The obtained fresh individual tumour tissues were immediately implanted subcutaneously in the flank area of SCID mice, subcutaneously, for establishment of PDX tumours, and the surplus fresh tissues were frozen at −80°C for analytical use or the frozen tumour tissue specimens were requested after the Roswell Park Pathology network froze the fresh tumour tissues. In summary, the de‐identification procedure includes the removal of tumour tissues with or without adjacent non‐tumour tissues during surgery and their subsequent procurement. The specimens that were examined by a pathologist have since been archived for various lengths of time. The examined specimens will be coded by the designated investigator(s), and no patient identifiers (protected health information) are available to the project PI and team members.

All in vivo studies were performed following the mouse protocol (1192 M) approved by the Institutional Animal Care and Use Committee at Roswell Park. The FL118 MTD for the weekly x 4 schedule was defined as 10 mg/kg for mice in our previous studies.^13^ For in vivo studies, 8‐ to 10‐week‐old female or male severe combined immunodeficiency (SCID) mice (20–25 g) were obtained from the Division of Laboratory Animal Resources, Roswell Park. Female SCID mice were housed at five mice per cage with water and food ad libitum. In this study, three types of human tumour models were used: human PDAC PDX tumour models, human CRC cell‐established tumour models and human DDX5 KO PDAC cell clone‐established xenograft tumour models. These human tumours were maintained in SCID mice. Experimental human tumour animal model setup: tumours maintained on SCID mice were isolated, and a piece of non‐necrotic tumour tissue (30–40 mg) was subcutaneously transplanted into the flank area of SCID mice. When human tumours grew to 100–200 mm^3^ (defined as Day 0), mice were randomly divided into the required groups (five mice per group) for oral administration of FL118 on a weekly x 4 (arrowed). Tumour establishment in SCID mice from CRC cell lines or genetically engineered PDAC cell pools or individual cell clones: Relevant CRC or PDAC cells (2 × 10^6^ per tumour site) were subcutaneously injected into each site in the flank area of SCID mice for 3–5 mice (dependent on the study need), and tumour growth was monitored and documented over time.

Tumour length (L) and width (W) were measured using digital Vernier callipers two to three times per week until the end of the experimental studies. The tumour volume (v) was calculated using the formula v = 0.5 (L x W^2^). Then, the tumour size was divided by the day 0 tumour size as a percentage of the tumour size versus day 0. The mean tumour volume ± standard deviation (SD) at each time point was derived from five mice in each group. The tumour curves were made using Microsoft Excel.

### 4.19 Statistical analysis

Protein band intensities from western blot results were quantified using ImageJ software, normalised to controls and presented as the relative intensity. Real‐time qRT‐PCR and MTT assay results were analysed using Microsoft Excel and presented as the mean ± SD derived from more than or equal to three independent assays. The statistical significance of differences was determined by Student's *t*‐test, with a *p* value of ≤ .05 considered significant. *p < .05; **p < .01; ***p < .001.

## CONFLICT OF INTEREST

FL118 and FL118 platform‐based analogues will be further developed in Canget BioTekpharma LLC (www.canget‐biotek.com), a Roswell Park Comprehensive Cancer Center spinoff company. Xiang Ling and Fengzhi Li are two of the 17 initial investors for the development of FL118 and FL118 core structure‐relevant anticancer agents. Other authors declare that they have no competing interests. Relevant patents include USA patent US 7,569,221 B2, 2009; PCT/US2011/058558, USA2011; and PCT/US2015/022095, USA2015.

## Supporting information

Supporting Information
