# Supplementary material for: FL118, acting as a ‘molecular glue degrader’, binds to dephosphorylates and degrades the oncoprotein DDX5 (p68) to control c‐Myc, survivin and mutant Kras against colorectal and pancreatic cancer with high efficacy
Source: Clin Transl Med. 2022 May 23;12(5):e881. doi: 10.1002/ctm2.881 (PMC9126027; doi:10.1002/ctm2.881)
Supplement: Supplementary file 1 — Supporting Information [file CTM2-12-e881-s001.pdf]

Supplemental materials

FL118 as a 'molecular glue degrader' binds to, dephosphorylates and degrades the oncoprotein DDX5 (p68) to control c-Myc, survivin and mutant Kras against colorectal and pancreatic cancer with high efficacy

Xiang Ling [1,2†*], Wenjie Wu [1,2†], Ieman A. M. Aljahdali [1,3], Jianqun Liao[2], Sreevidya Santha[2], Christos Fountzilas [4,5], Patrick M Boland [4‡] and Fengzhi Li [1,5*]

*Corresponding authors. E-mail: fengzhi.li@roswellpark.org and Xiang.Ling@RoswellPark.org

Supplemental Text Methods

Replacement of the Tris-HCl buffer with the PBS buffer for purified proteins for drug-protein binding analyses using isothermal titration calorimetry (ITC)

After the purified proteins were eluted from the agarose resin with 3X flag peptide, the same purified protein samples from different batches were combined. Then, the DDX5 or Top1 proteins in the 1X wash buffer (50mM Tris-HCl, 150mM NaCl, pH7.4) containing Flag peptide were replaced by using 1.5 mL size Nanosep 10K Omega device (PALL Life Sciences). Briefly, 200-300 µL purified proteins with a concentration of 1-2 µg/µL were pipetted into the sample reservoir of the Nanosep device and centrifuged at 8000 RPM for 15-20 min to remove the 1X wish buffer with Flag peptides (removing the flow-out after each time centrifugation). Then, the retained proteins were washed with 300-400 µL 1X phosphate-buffered saline (PBS) (137 mM NaCl, 10 mM Phosphate, 2.7 mM KCl at pH7.4) 4 times by centrifugation as above. The PBS-washed proteins were then resuspended in 200-300 µL 1X PBS (pH7.4) depending on the initial protein concentration. The PBS-resuspended protein samples were recovered from the reservoir and the protein concentrations were determined using the nano-drop reader (Bio-Rad) and BCA Protein Assay (Thermo/Pierce) for double-making sure the concentration. This buffer replacement is important for the next step of the isothermal titration calorimetry (ITC) to analyze FL118 binding affinity to the proteins.

Methods to determine the minimal DMSO concentration required for FL118 at 100 µM without precipitation to match the requirement for FL118-protein binding analysis by ITC

This includes several steps. **(1)** <u>Preparation of required materials and reagents</u>: (**a**) 1x PBS (137 mM NaCl, 10 mM Phosphate, 2.7 mM KCl at pH7.4); (**b**) 2 mM cold FL118 in DMSO (cold FL118, 1.1mg +1,403 µL DMSO); and (**c**) ^{3}H-FL118 stock (the dry ^{3}H-FL118 ,5.6 Ci/mmol, was

dissolved in 446 µl DMSO to make a solution of 11.2 mCi/ml, which has the ^{3}H-FL118 concentration of 11.2 mCi/ml / 5.6 Ci/mmol x 1000 = 2000 µM/2 mM). **(2)** Prepared a FL118 (cold) and ^{3}H-FL118 mix with the ratio of 9:1 at 2 mM level for the final concentration of FL118/^{3}H-FL118 at 100 µM with 5% DMSO: 225 µl 2 mM cold FL118 were mixed with 25 µl 2 mM ^{3}H-FL118 to make 250 µl (2 mM). Then, 10 µL 2 mM FL118/^{3}H-FL118 were added into 190 µl 1xPBS to make 200 µl to make FL118 at 100 µM containing 5% DMSO. Of note, took out 20 µl before centrifuge as pre-Spin control. **(3)** Prepared a FL118 and ^{3}H-FL118 mix with the ratio of 9:1 at 1.25 mM level for the final concentration of FL118/^{3}H-FL118 at 100 µM containing 8% DMSO: Took 62.5 µl (2 mM FL118/^{3}H-FL118 mix) + 37.5 µl DMSO to make 100 µL (FL118, 1.25 mM). Then, added 16 µl 1.25 mM FL118/^{3}H-FL118 into 184 µl 1xPBS to make 200 µL FL118 100 µM containing 8% DMSO. Of note, took out 20 µl before centrifuge as pre-Spin control. **(4)** Prepared a FL118 and ^{3}H-FL118 mix with the ratio of 9:1 at 1 mM level for the final concentration of FL118/^{3}H-FL118 at 100 µM with 10% DMSO: Took 150 µl (2 mM FL118/^{3}H-FL118 mix) + 150 µl DMSO to make 300 µl (FL118, 1 mM). Then, added 20 µl 1 mM FL118/^{3}H-FL118 into 180 µl 1xPBS to make 200 µl FL118 at 100 µM containing 10% DMSO. Of note, took out 20 µl before centrifuge as pre-Spin control. **(5)** Prepared a FL118 and ^{3}H-FL118 mix with the ratio of 9:1 at 0.625 mM level for the final concentration of FL118/^{3}H-FL118 at 100 µM with 16% DMSO: Took 20 µl (1.25 mM FL118/^{3}H-FL118 mix) + 20 µl DMSO to make 40 µl (FL118, 0.625 mM). Then, added 32 µl 0.625 mM FL118/^{3}H-FL118 into 168 µl 1xPBS to make 200 µl FL118 at 100 µM containing 16% DMSO. Of note, took out 20 µl before centrifuge as pre-Spin control. **(6)** Prepared a FL118 and ^{3}H-FL118 mix with the ratio of 9:1 at 0.5 mM level for the final concentration of FL118/^{3}H-FL118 at 100 µM with 20% DMSO: Take 150 µl (1 mM FL118/^{3}H-FL118 mix) + 150 µl DMSO to make 300 µl (0.5 mM). Then, added 40 µl 0.5 mM FL118/^{3}H-FL118 into 160 µl 1xPBS to make 200 µl FL118 a t100 µM containing 20% DMSO. Of note, took out 20 µl before centrifuge as pre-Spin control. **(7)** Prepared a FL118 and ^{3}H-FL118 mix with the ratio of 9:1 at 0.4 mM level for the final concentration of FL118/^{3}H-FL118 at 100 µM with 25% DMSO: Took 15 µl (2 mM FL118/^{3}H-FL118 mix) + 60 µl DMSO to make 75 µl (0.4 mM). Then, added 50 µl 0.4 mM FL118/^{3}H-FL118 into 150 µl 1xPBS to make 200 µl FL118 at 100 µM containing 25% DMSO. Of note, took out 20 µl before centrifuge as pre-Spin control. **(8)** Prepared a FL118 and ^{3}H-FL118 mix with the ratio of 9:1 at 0.25 mM level for the final concentration of FL118/^{3}H-FL118 at 100 µM with 40% DMSO: Took 150 µl (0.5 mM FL118/^{3}H-FL118 mix) + 150 µl DMSO to make 300 µl (0.25 mM). Then, added 80 µl 0.25 mM FL118/^{3}H-FL118 into 120 µl 1xPBS to make 200 µl FL118 at 100 µM containing 40% DMSO. Of note, took out 20 µl before centrifuge as pre-Spin control.

After took out 20 µl solution from each of the above-prepared solutions as controls, then centrifuged all seven 1.5 ml tube at 3,000 rpm for 1 min, and then took out another 20 µl supernatant from each of the seven tubes. The seven 1.5 ml tube were further centrifuged at 12,000 rpm for 1 min, and then took out another 20 µl supernatant from each 1.5 ml tube. Each of the 20 µL were put into a scintillation vial containing 3 ml scintillation solution for tritium counting, and the results are shown in the **Table S2**.

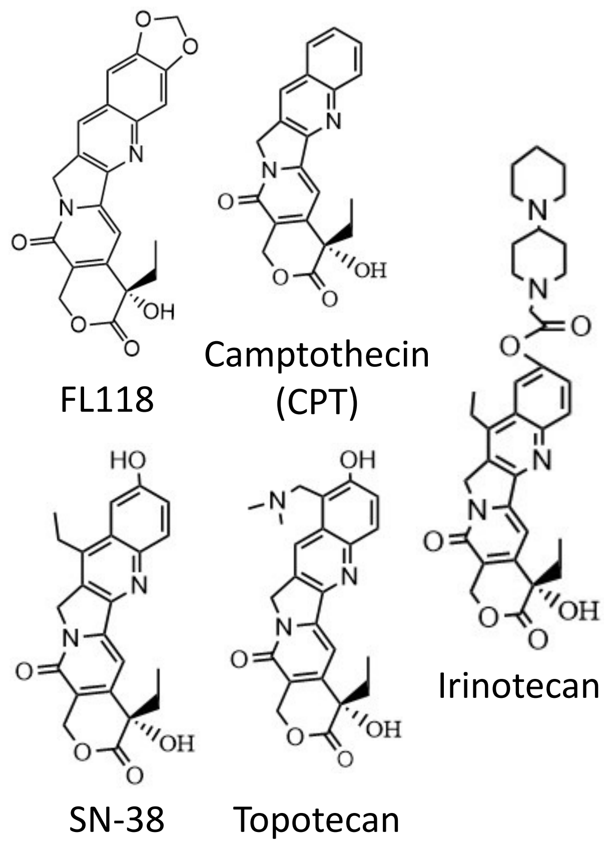

Fig. S1. The chemical structure of FL118, camptothecin (CTP), and CPT analogues, irinotecan (CPT-11), SN-38 (active metabolite of irinotecan) and topotecan.

A. An example of the "Mannich Reaction"

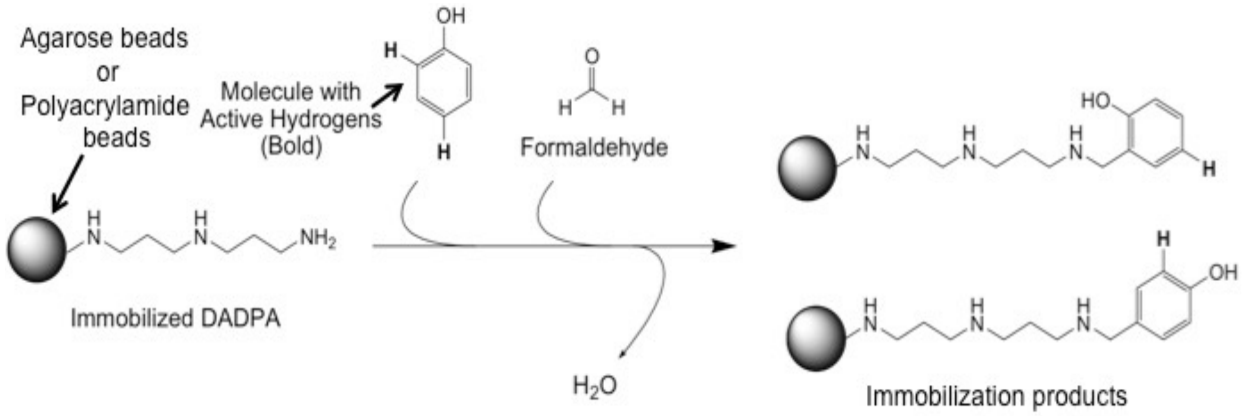

B. FL118-protein interaction testing using NonoSep Omega Centrifugal Devices (PALL, Life Sciences)

Proteins on 3K NanoSep Device	Tritium (^{3}H) CPM counts in retained parts
BSA	3,877 ± 550
Top1	13,322 ± 1600
DDX5	123,763 ± 6800

Fig. S2. A. An example of the "Mannich Reaction". **B.** The interaction of FL118 with bovine serum albumin (BSA), topoisomerase 1 (Top1) and DDX5. Counts per minute (CPM) is the mean ± SD derived from three-time independent testing. The detailed method is provided in the Section 4.6 (Determination of FL118-DDX5 and FL118-Top1 binding using Nanosep Device) in the paper.

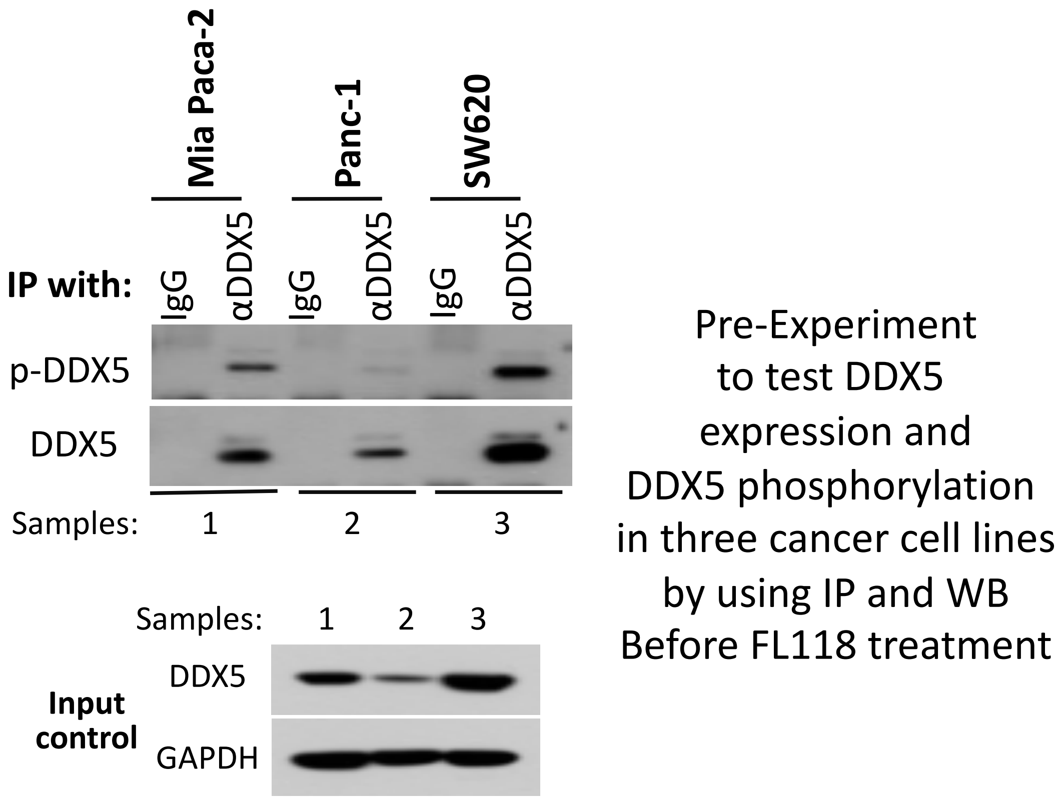

Fig. S3. Determination of DDX5 tyrosine (Tyr/Y) phosphorylation and expression in multiple CRC/PDAC cell lines. Sub-confluently growing Mia Paca-1, Panc-1 and SW620 cells without FL118 treatment were lysed and performed immunoprecipitation (IP) with anti-DDX5 antibody (αDDX5), followed by Western blots using Tyr/Y-phospho-specific antibody and DDX5 antibody, respectively. The input control is the 10% of cell lysates before IP for Western blots with DDX5 and GAPDH antibodies. GAPDH is the internal control for total protein loading.

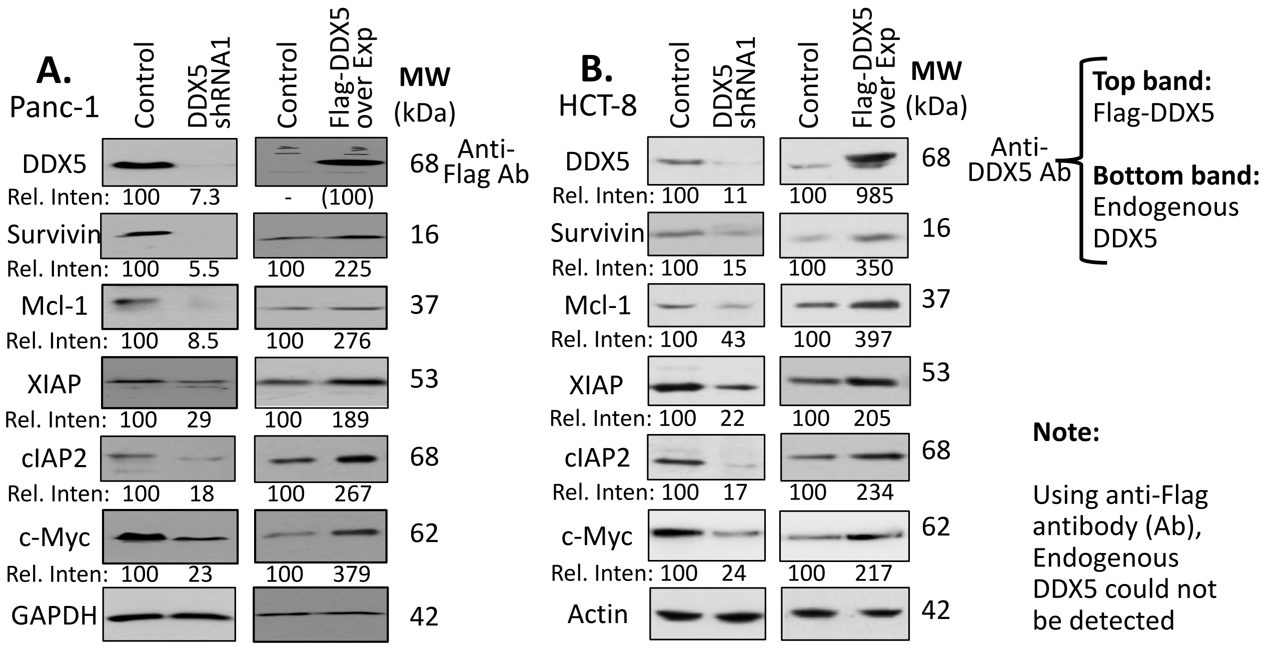

Fig. S4. A., **B.** (left panels): ShRNA silencing of DDX5 decreases the expression of survivin, Mcl-1, XIAP, cIAP2, and/or c-Myc in different degrees. Cells were infected with control or DDX5 shRNAs as shown. Infected cells were lysed 48h post infection; the cell lysates were used to determine the expression of DDX5, survivin, Mcl-1, XIAP, cIAP2, and/or c-Myc through Western blots. **A.**, **B.** (right panels): Forced expression of flag-DDX5 increases the expression of survivin, Mcl-1 XIAP, cIAP2, and/or c-Myc in different degrees. Cells were transfected with empty vector (control) or flag-DDX5 expression vectors (RC200371, OriGene) using Lipofectamine 2000. Cells were lysed 48h post-transfection and subjected to Western blot analysis using their corresponding antibodies. Overexpressed flag-DDX5 was detected using flag antibody in (A); endogenous DDX5 could not be detected in this case (A, right panel). Overexpressed flag-DDX5 was detected using DDX5 antibody in (B); in this case, flag-DDX5 protein is slightly larger (B, right panel). Relative intensities (Rel. Inten) of the Western blot bends were marked out by setting the control band as 100 after normalized to the internal controls (GAPDH for A and Actin for B).

DDX5 shRNA1 (V2LHS_24065, location 172_0624-E -11)
Hairpin sequence color codes - **mir-30 context** sense loop antisense

5'-TGCTGTTGACAGTGAGCGCACACTTTCTTTACACCTAATA
TAGTGAAGCCACAGATGTATATTAGGTGTAAAGAAAGTGT
ATGCCTACTGCCTCGGA-3'

DDX5 shRNAs2 (V2LHS_24063, location 172_0574-G -12)
Hairpin sequence color codes - **mir-30 context** sense loop antisense

5'-TGCTGTTGACAGTGAGCGACGTGACTGGGTTCTAAATGAA
TAGTGAAGCCACAGATGTATTCATTTAGAACCCAGTCACG
CTGCCTACTGCCTCGGA-3'

Fig. S5. DDX5-specific shRNA DNA sequences. Two DDX5-specific shRNA DNA sequences used in this study are shown.

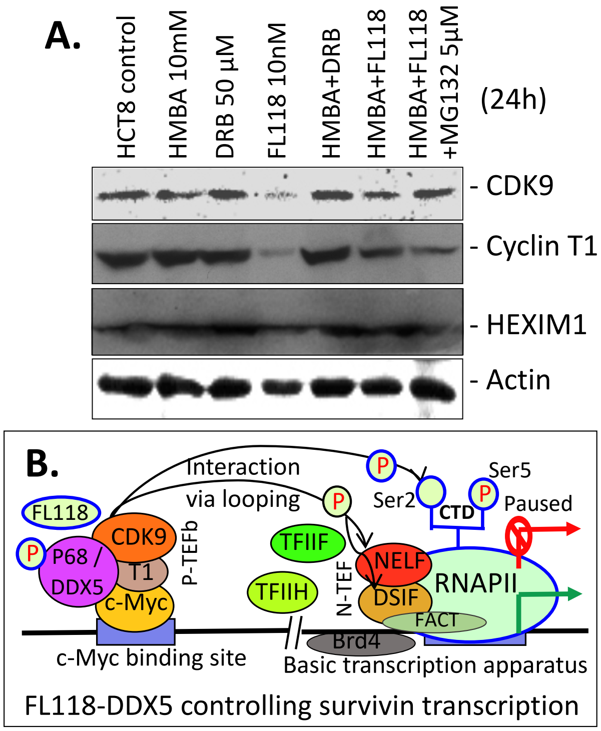

Fig. S6. A. FL118 inhibits the expression of CDK9 and T1: HCT8 CRC cells were treated with and without various reagents for 24h as shown, followed by WB analysis of the expression for CDK9 and T1. Actin is an internal control. Note: **HMBA** (hexamethylene bisacetamide), a P-TEFb activator; **DRB** (5,6-dichloro-1-b-D-ribofuranosyl-benzimidazole), a P-TEFb activity inhibitor. **B.** Preliminary working model for DDX5 to turn on survivin transcription: Constitutively activated DDX5 interacts with TF (c-Myc in this model) to recruit P-TEFb to form a complex on the survivin promoter, which activates CDK9 to interact with and phosphorylate relevant proteins to promote transcriptional elongation (**TE**) as shown by a green arrow *(65)*. This includes phosphorylation of the CTD (C-terminal domain) of RNAPII at the Ser2 position and the **N-TEF** (negative transcription elongation factor), which consists of **NELF** (negative elongation factor) and **DSIF** (DRB-sensitivity inducing factor). This in turn triggers TFIIF and/or TFIIH to replace NELF as well as recruitment of **Brd4** (a **br**omo**d**omain-containing protein involving in chromatin targeting) and **FAST** (facilitates chromatin transcription) *(67, 81, 82)*. As a result, the RNAPII complex will transit its **paused state** into its **TE state** to promote survivin pre-mRNA transcription and maturation *(66)*. However, inhibition of DDX5 by FL118 would disrupt this process.

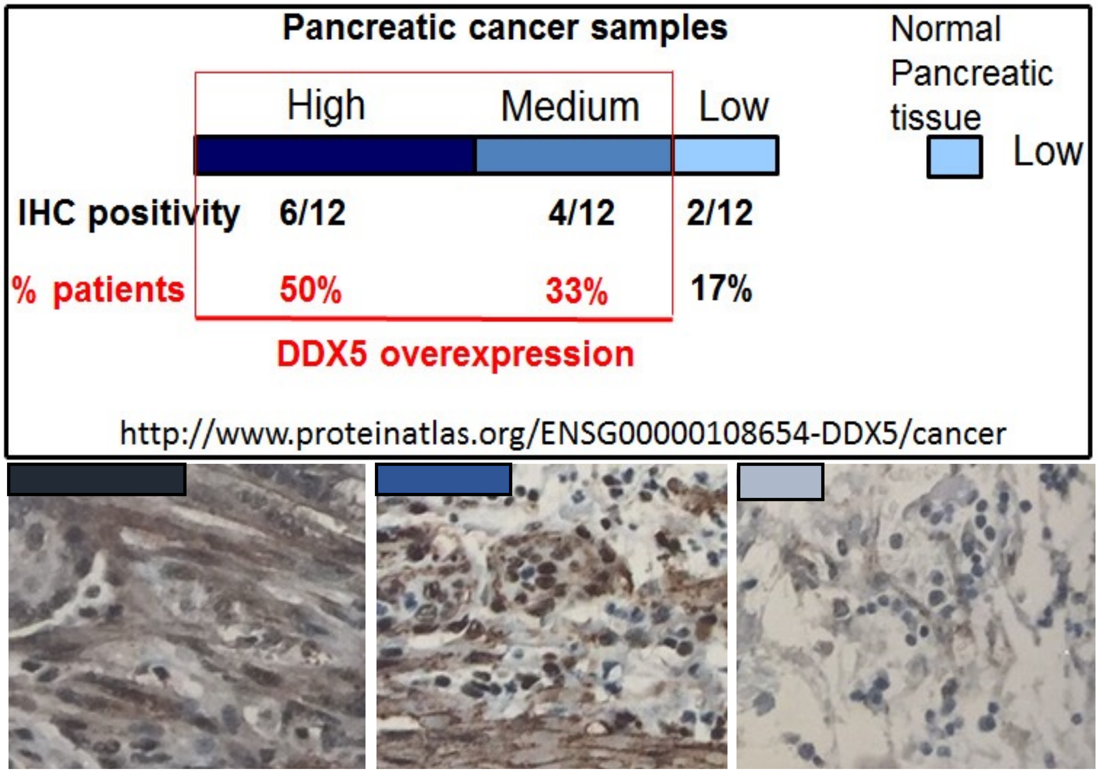

Fig. S7 DDX5 expression is enhanced in PDAC tumor tissues: The Human Protein Atlas data indicate that DDX5 expression is enhanced in 83% of PDAC tumors.

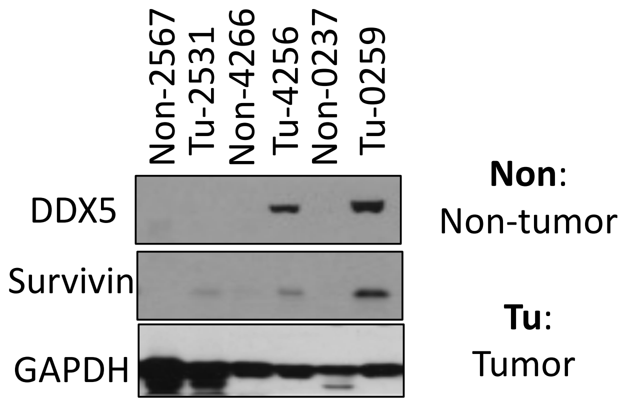

Fig. S8 Western blots show DDX5 and survivin expression in 3 pairs of clinical CRC tumor specimens, GAPDH in is the internal control. As shown, high DDX5 is associated with high survivin.

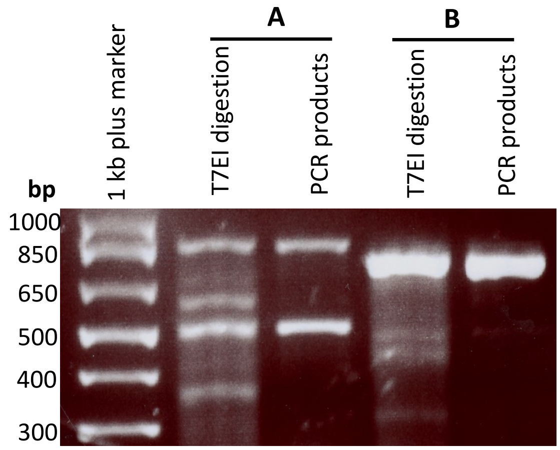

Fig. S9. Results from the detection of DDX5 KO cells in the KO cell pool. **A.** PCR products of primer pair A (Cas9_DDX5_AA_AB-F and Cas9_DDX5_AA_AB-R). **B:** PCR products of primer pair B (Cas9_DDX5_AA_AC-F: 5' and Cas9_DDX5_AA_AC-R). The principle of T7EI assay to determine whether there are DDX5 gene KO cells in the pool is that: Use PCR to amplify the targeted genomic region of the DDX5 gene modified region. Then, denature and reanneal PCR products in a thermal cycler to allow potential heteroduplex formation after the reannealing process between wild-type and CRISPR–mutated DNA. Then, digest reannealed PCR products with T7EI enzyme, which cleaves mismatched DNA heteroduplexes. If there are heteroduplex formation (i.e., existence of DDX5 KO cells), after digestion, other small DNA bands would be detected. Otherwise, if no KO cell exists, T7EI enzyme-digested and undigested PCR products will be the same results of no changes for the DNA band pattern shown on the get after electrophoresis.

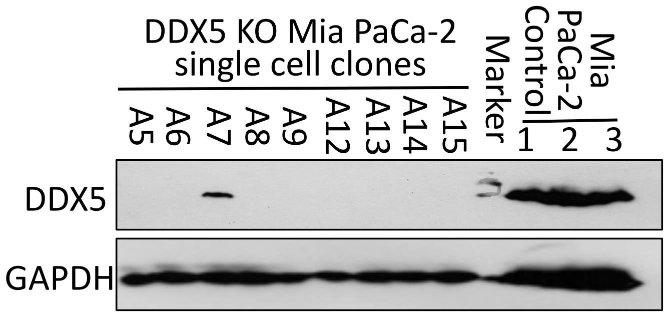

Fig. S10. Detection of DDX5 expression in the Mia Paca-2 single cell clones with DDX5 knockout (KO) generated by using the vector-free CRISPR technology. The expression profile of DDX5 in various Mia Paca-2 individual cell clones in parallel with the non-DDX5 KO control Mia Paca-2 cells analyzed by using Western blots is shown. GAPDH is the internal control.

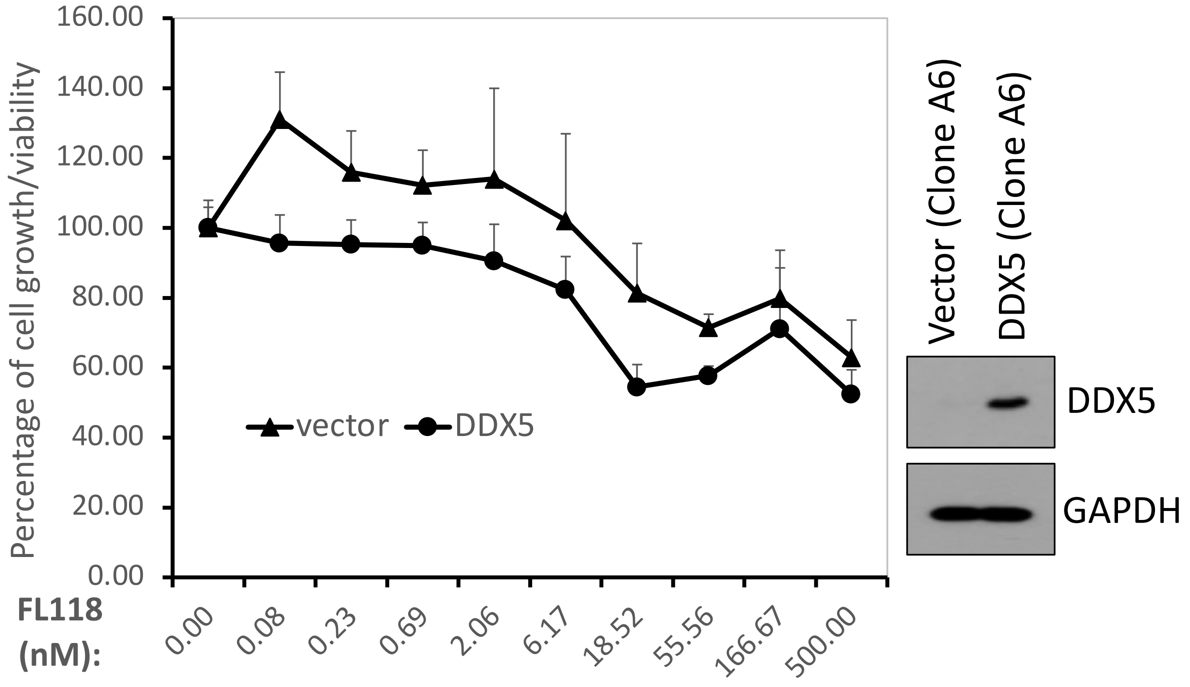

Fig. S11. Determination of the Mia Paca-2 DDX5 KO A6 clone cells' sensitivity to FL118 treatment with and without re-expression of DDX5. After transfection of control vector (pCMV6-Entry) and DDX5 expression vector (pCMV6-Entry-DDX5) into Mia Paca-2 DDX5 KO A6 clone cells with a limited kanamycin selection. A part of the DDX5 KO A6 clone transfectant cells were analyzed for DDX5 re-expression using Western blots (the result in right panel). GAPDH is the internal control. Another part of the DDX5 KO A6 clone transfectant cells were seeded in 96-well plated overnight and then treated on the following day with a series of FL118 concentration as shown for 72h. Cell viability/growth were then analyzed using MTT assay. Each curve is the mean + SD derived from 3 independent tests (the result in left panel).

Table S1. Results of ^{3}H-FL118-DEAD box protein binding

Database ID	Ultimate ORF ID	Description [Use of tritium (3H)-labeled FL118 as a probe]	Negative Z-Score*	FL118 Z-Score*
NM_004939.1	IOH14578	DEAD (Asp-Glu-Ala-Asp) box polypeptide 1 (DDX1)	0.72833	0.17385
BC047455.1	IOH26531	DEAD (Asp-Glu-Ala-Asp) box polypeptide 4 (DDX4)	0.29952	0.02775
BC039826.1	IOH26225	DEAD (Asp-Glu-Ala-Asp) box polypeptide 6 (DDX6)	0.78491	-0.53613
NM_004397.3	IOH45655	Probable ATP-dependent RNA helicase DDX6	-4.22936	-0.3412
NM_004398.2	IOH38427	DEAD (Asp-Glu-Ala-Asp) box polypeptide 10 (DDX10)	0.25449	-0.01765
BC050522.1	IOH26996	DEAD/H (Asp-Glu-Ala-Asp/His) box polypeptide 11 (DDX11)	-1.95948	0.06313
NM_030881.2	IOH1750	DEAD (Asp-Glu-Ala-Asp) box polypeptide 17 (DDX17)	0.42192	0.20888
BC003360.1	IOH2892	DEAD (Asp-Glu-Ala-Asp) box polypeptide 18 (DDX18)	0.02568	1.41679
NM_007242.3	IOH3925	DEAD (Asp-Glu-Ala-As) box polypeptide 19B (DDX19B)	0.03888	-0.15213
BC031062.1	IOH27218	DEAD (Asp-Glu-Ala-Asp) box polypeptide 20 (DDX20)	0.06196	-0.10666
BC011556.1	IOH13000	DEAD (Asp-Glu-Ala-Asp) box polypeptide 20 (DDX20)	-0.08376	0.76704
NM_004728.2	IOH46173	DEAD (Asp-Glu-Ala-Asp) box polypeptide 21 (DDX21)	0.80949	-0.76238
NM_013264.2	IOH45462	DEAD (Asp-Glu-Ala-Asp) box polypeptide 25 (DDX25)	-0.20313	0.89783
NM_005804.2	IOH3477	DEAD (Asp-Glu-Ala-Asp) box polypeptide 39 (DDX39)	0.64786	1.03613
NM_138998.1	IOH21408	DEAD (Asp-Glu-Ala-Asp) box polypeptide 39 (DDX39)	0.38945	-0.43721
BC015505.1	IOH9981	DEAD (Asp-Glu-Ala-Asp) box polypeptide 42 (DDX42)	0.23496	-0.32012
BC066938.1	IOH40083	DEAD (Asp-Glu-Ala-Asp) box polypeptide 43 (DDX43)	-1.41173	-0.03906
NM_016355.3	IOH39973	DEAD (Asp-Glu-Ala-Asp) box polypeptide 47 (DDX47)	-0.04594	-2.638
BC000979.2	IOH2932	Probable ATP-dependent RNA helicase DDX49	-0.71964	-0.09828
NM_019070.1	IOH5264	DEAD (Asp-Glu-Ala-Asp) box polypeptide 49 (DDX49)	-0.85024	1.28934
BC001132.1	IOH3853	DEAD (Asp-Glu-Ala-Asp) box polypeptide 54 (DDX54)	0.74757	1.18306
BC030020.2	IOH22410	DEAD (Asp-Glu-Ala-Asp) box polypeptide 55 (DDX55)	0.74673	0.66028
BC035911.1	IOH25946	DEAD (Asp-Glu-Ala-Asp) box polypeptide 55 (DDX55)	0.73311	0.49841
NM_014314.3	IOH52971	Probable ATP-dependent RNA helicase DDX58	-0.0081	0.28653
NM_004396.5	IOH1655	DEAD/H (Asp-Glu-Ala-Asp/His) box polypeptide 5 (DDX5)	0.27649	8.30128
NM_002046.2	IOH3380	glyceraldehyde-3-phosphate dehydrogenase (GAPDH)	-0.20833	-0.3624
NM_003536.2	IOH6208	Histone H3	-0.22304	-1.16182

*****Z-score is a protein-binding tritium (^{3}H) signal subtracted from the mean signal from all the human proteins tested on the protein microarray, then divided by the standard deviation (**SD**) of all proteins. A Z-Score greater than 2.0 means a signal greater than 2x SD above the median signal from all the human proteins tested on the ProtoArray. Negative Z-score is <1. **Note:** DDX5 is Flag-tagged, and all other proteins on the microarray are GST-tagged.

Table S2. Use of centrifugation to determine the minimal DMSP concentration that does not induce FL118 precipitation at 100 µM

**Sample # (FL118)**	**DMSO %**	**Sample**	**Spin for 1 min**	**DPM/CPM**
1 (FL118 100µM)	5%, 1-1	20 µl	Pre-Spin	101893/29099
2 (FL118 100µM)	5%, 2-2	20 µl	After Spin at 3k rpm	93968/26440
3 (FL118 100µM)	5%, 3-3	20 µl	After Spin at 12k rpm	89487/25386
4 (FL118 100µM)	8%, 4-1	20 µl	Pre-Spin	101673/28696
5 (FL118 100µM)	8%, 5-2	20 µl	After Spin at 3k rpm	99447/28481
6 (FL118 100µM)	8%, 6-3	20 µl	After Spin at 12k rpm	104522/29542
7 (FL118 100µM)	10%, 7-1	20 µl	Pre-Spin	107627/30810
8 (FL118 100µM)	10%, 8-2	20 µl	After Spin at 3k rpm	104440/29105
9 (FL118 100µM)	10%, 9-3	20 µl	After Spin at 12k rpm	105432/30273
10 (FL118 100µM)	16%, 10-1	20 µl	Pre-Spin	111074/31108
11 (FL118 100µM)	16%, 11-2	20 µl	After Spin at 3k rpm	109799/30973
12 (FL118 100µM)	16%, 12-3	20 µl	After Spin at 12k rpm	110081/30902
13 (FL118 100µM)	20%, 13-1	20 µl	Pre-Spin	101766/29215
14 (FL118 100µM)	20%, 14-2	20 µl	After Spin at 3k rpm	105522/29997
15 (FL118 100µM)	20%, 15-3	20 µl	After Spin at 12k rpm	105776/30137
16 (FL118 100µM)	25%. 16-1	20 µl	Pre-Spin	134269/38410
17 (FL118 100µM)	25%, 17-2	20 µl	After Spin at 3k rpm	138718/39547
18 (FL118 100µM)	25%, 18-3	20 µl	After Spin at 12k rpm	139932/40121
19 (FL118 100µM)	40%, 19-1	20 µl	Pre-Spin	138588/39897
20 (FL118 100µM)	40%, 20-2	20 µl	After Spin at 3k rpm	130401/37725
21 (FL118 100µM)	40%, 21-3	20 µl	After Spin at 12k rpm	144315/42167

Conclusions from the result shown in Table S2: This centrifugation testing found that 5% DMSO is not good enough, but 8% DMSO is good enough to maintain FL118 at 100 µM concentration in 1xPBS without precipitation (i.e., a true solution without FL118 precipitation at 8% DMSO). Of note, the DMSO at ≤10% in the protein-small molecule solution for isothermal titration calorimetry (**ITC**) determination of the small molecule binding affinity to the protein is the best. DMSO > 10% will be a sub-optimal concentration for ITC studies of drug-protein interaction affinity.
